# Efficient and regioselective synthesis of dihydroxy-substituted 2-aminocyclooctane-1-carboxylic acid and its bicyclic derivatives

**DOI:** 10.3762/bjoc.18.7

**Published:** 2022-01-06

**Authors:** İlknur Polat, Selçuk Eşsiz, Uğur Bozkaya, Emine Salamci

**Affiliations:** 1 Department of Chemistry, Faculty of Sciences, Atatürk University, 25240 Erzurum, Turkey; 2 Hakkari University, Vocational School of Health Services, Department of Medical Services and Techniques, 30000 Hakkari, Turkey; 3 Department of Chemistry, Hacettepe University, 06800 Ankara, Turkey

**Keywords:** aminocyclitol, azidolysis, bicyclic β-lactam, bicyclic lactone, cyclic β-amino acids, DFT

## Abstract

The first synthesis of 2-amino-3,4-dihydroxycyclooctane-1-carboxylic acid, methyl 6-hydroxy-9-oxo-8-oxabicyclo[5.2.1]decan-10-yl)carbamate, and 10-amino-6-hydroxy-8-oxabicyclo[5.2.1]decan-9-one starting from *cis*-9-azabicyclo[6.2.0]dec-6-en-10-one is described. *cis*-9-Azabicyclo[6.2.0]dec-6-en-10-one was transformed into the corresponding amino ester and its protected amine. Oxidation of the double bond in the *N*-Boc-protected methyl 2-aminocyclooct-3-ene-1-carboxylate then delivered the targeted amino acid and its derivatives. Density-functional theory (DFT) computations were used to explain the reaction mechanism for the ring opening of the epoxide and the formation of five-membered lactones. The stereochemistry of the synthesized compounds was determined by 1D and 2D NMR spectroscopy. The configuration of methyl 6-hydroxy-9-oxo-8-oxabicyclo[5.2.1]decan-10-yl)carbamate was confirmed by X-ray diffraction.

## Introduction

**Figure 1:**
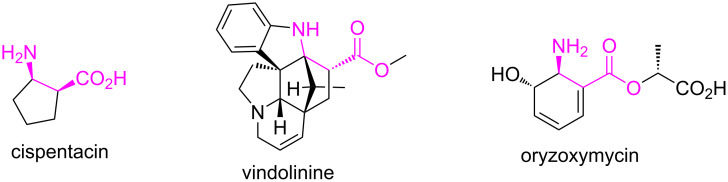
Examples of natural products containing β-amino acids.

Cyclic β-amino acids have for the past few decades aroused widespread synthetic interest owing to their diverse biological activities, especially applications in the field of medicinal chemistry. β-Amino acids (i.e., amino acids containing an extra methylene group in the backbone) occur naturally in peptidic structures [1–5] and have been used in peptide design to obtain mixed peptides that retain their biological activities [6–7]. Moreover, they can be used as starting substances for different heterocycles, as precursors for the synthesis of polymers, as potential pharmacons, for the synthesis of natural products or analogues, and also as building blocks in drug research [8–11]. Furthermore, some β-amino acid derivatives have antibiotic (oryzoxymycin) and antifungal activities (Figure 1) [12–13].

Among the cyclic β-amino acids, the most widely investigated derivatives are the five- and six-membered derivatives [8–10,14–15], but only a few synthetic methods are available for the synthesis of β-amino acids containing seven- [14–16], and eight- [14,17–18] membered rings. Only one of these studies previously reported by Fülöp and co-workers was on the synthesis of hydroxylated cyclooctane amino acids starting from 1,5-cyclooctadiene [17], the others were on the synthesis of non-hydroxylated cyclooctane amino acids [14,18]. Also in other ring systems, only non-hydroxylated cyclic amino acids and derivatives were synthesized [8–10,15–16]. Therefore, we were inspired to develop new methods for the synthesis of hydroxylated β-amino acid derivatives containing eight-membered rings. We have recently reported the synthesis of various eight-membered aminocyclitols and their derivatives [19–25]. In the present paper, we describe the synthesis of some hydroxylated β-amino acid derivatives containing eight-membered rings starting from *cis*,*cis*-1,3-cyclooctadiene.

## Results and Discussion

**Scheme 1:**
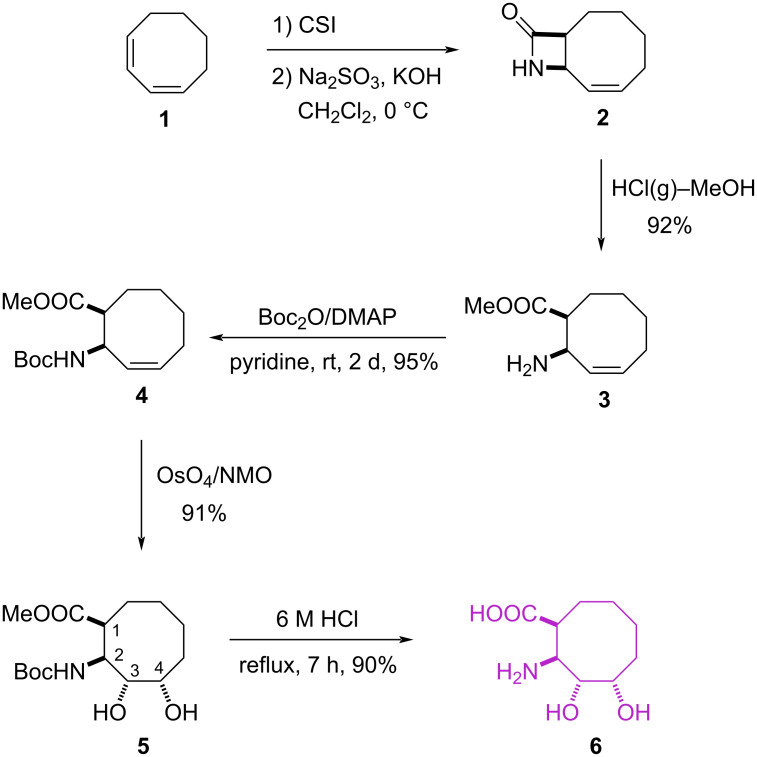
Synthesis of cyclic β-amino acid **6**.

Initially, we focused on the synthesis of β-lactam **2**, which was prepared by the cycloaddition of chlorosulfonyl isocyanate (CSI) to *cis*,*cis*-1,3-cyclooctadiene, as described in the literature [26]. β-Lactam **2** was transformed into *cis*-amino ester **3** by cleavage of the lactam ring with HCl(g) in MeOH (Scheme 1).

*N*-Boc protection of *cis*-amino ester **3** with (Boc)_2_O in pyridine and 4-(dimethylamino)pyridine (DMAP) gave *N*-Boc-amino ester **4** (yield 95%). The ^1^H and ^13^C NMR spectroscopic data of **4** were in agreement with the proposed structure. Treatment of **4** with OsO_4_/NMO gave the expected diol **5** as a single isomer in 91% yield. We assume that the *trans* selectivity of hydroxylation in ester **4** is due to the steric effect of the presence of the bulky Boc group. The structure of **5** was determined with the help of 1D (^1^H and ^13^C) and 2D (COSY and HMQC) NMR spectra. The diagonal peak at 4.10 ppm has cross peaks with the protons resonating at 2.96, 3.95, and 5.39 ppm, respectively, in the COSY spectrum. The cross peak between H-2 and H-3/H-4 suggests that the proton H-2 should have a *trans* configuration relative to the proton H-3. Removal of the Boc protection by HCl resulted in the formation of the target β-amino acid **6**, which was characterized based on its NMR spectra (Scheme 1).

**Scheme 2:**
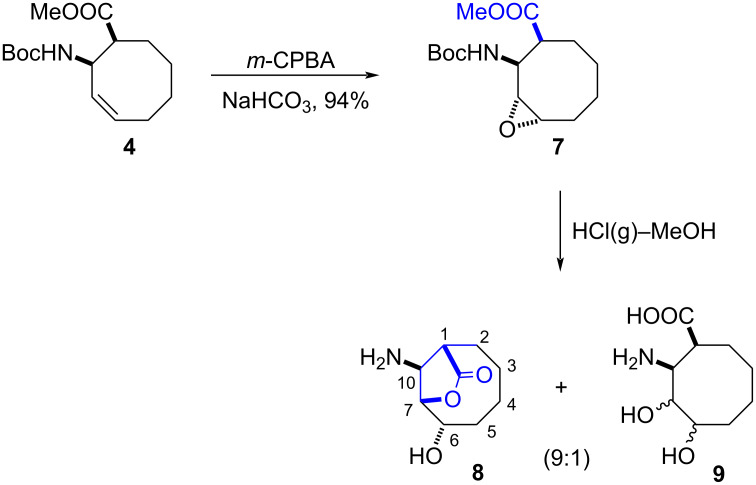
Epoxidation of Boc-protected amino ester **4** and hydrolysis of epoxide **7** with HCl(g)–MeOH.

The *N*-Boc-amino ester **4** was reacted with *m*-CPBA to give epoxide **7** as the sole product in 94% yield (Scheme 2). The structure of **7** was assigned based on its NMR spectra. Epoxide **7** was used as the precursor material in the synthesis of the other isomer of β-amino acid **6**. The ring-opening reaction of **7** with HCl(g) in MeOH resulted in a mixture of products **8** and **9** in a 9:1 ratio (^1^H NMR). The product **8** in the reaction mixture was purified by recrystallization from ethanol/ether, but all attempts to purify the expected product **9** failed. The product **8** was obtained as the major product in 80% yield, and the expected product **9** was formed as the minor product in 4% yield. We propose that diol isomer mixture **9** can be formed by solvolysis. The presence of the lactone ring in **8** was determined by 2D NMR spectroscopic data (COSY and HMQC). The diagonal peak at 4.62 ppm has cross peaks with the protons resonating at 4.26 and 4.39 ppm, respectively, in the COSY spectra of **8**. The cross peak between H-7 and H-6 showed strong correlation, which clearly supports the *trans* relation of the proton H-6. The cross peak between H-7 and H-10 also showed weak correlation, which clearly supports the *cis* relation of the proton H-10. In this reaction, lactonization and hydrolysis of the Boc group to the corresponding amine were observed. The formation of lactone **8** as the major product can be explained by nucleophilic attack of the neighbouring carboxyl group, which was formed by hydrolysis of the corresponding methyl ester.

**Scheme 3:**
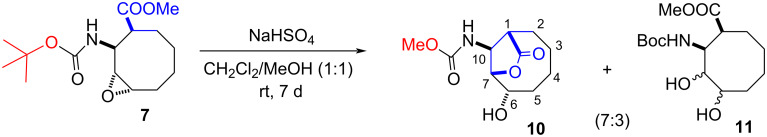
Reaction of epoxide **7** with NaHSO_4_ in methylene chloride/MeOH.

**Figure 2:**
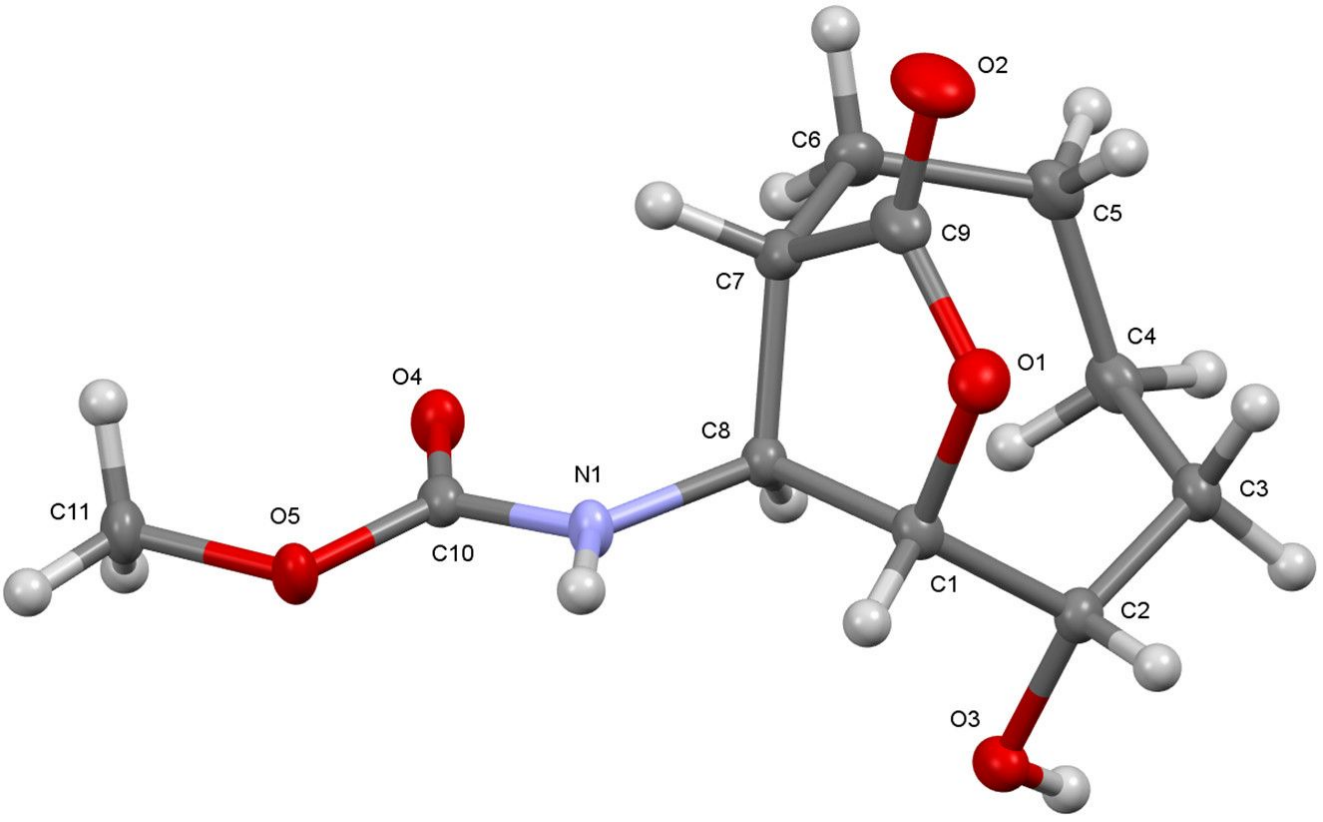
The X-ray crystal structure of **10**.

For the synthesis of other isomeric β-amino acid derivatives, epoxide **7** was treated with two equivalent amounts of NaHSO_4_ [27] in methylene chloride/MeOH at room temperature (Scheme 3). The formation of a mixture of products **10** and **11** in a 7:3 ratio was determined by NMR spectroscopy. The reaction mixture was purified using preparative silica gel TLC on a chromatotron with ethyl acetate/hexane (50:50) as the eluent to give carbamate **10** and diol isomer mixture **11** in 65% and 25% yields, respectively. However, all attempts to isolate isomer mixture **11** failed. Again, we suggest that diol isomer mixture **11** can be formed by solvolysis. The presence of the lactone ring in **10** was determined by 2D NMR spectroscopic data (COSY and HMQC). The diagonal peak at 4.19 ppm has cross peaks with the protons resonating at 4.49, 1.53, and 2.02 ppm, respectively, in the COSY spectra of **10**. The cross peak (δ 4.49 ppm) between H-6 and H-7 showed a strong correlation, which clearly supports the *trans* relation of the proton H-7. Furthermore, its structure was unambiguously confirmed by single crystal X-ray analysis (Figure 2) [28]. The formation of lactone **10** can again be explained by participation of the neighbouring group, as discussed above. However, during the purification on silica gel of the lactone-Boc product, its transesterification also resulted in corresponding methyl carbamate **10**.

**Scheme 4:**
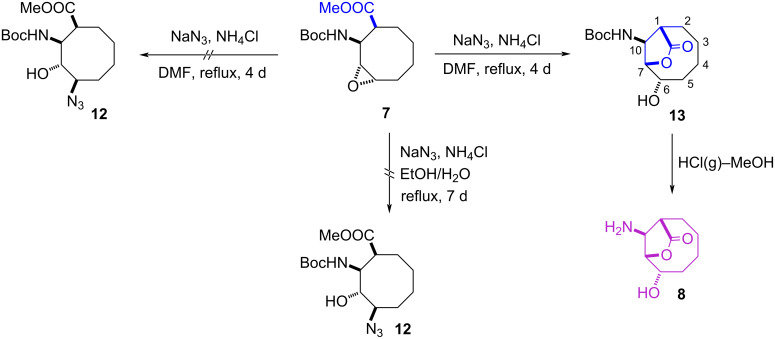
Synthesis of cyclic β-amino acid derivative **8**.

We then investigated the epoxide-ring-opening reaction of **7** with sodium azide, to introduce an extra amino group in position 4 on the cyclooctane skeleton. For this, epoxide **7** was treated with NaN_3_ in the presence of NH_4_Cl/DMF and this formed lactone **13** as the sole product in 80% yield (Scheme 4). The epoxide **7** was treated with NaN_3_ and NH_4_Cl/DMF to obtain compound **12**. From this reaction, the formation of unexpected lactone **13** was observed. The reaction was repeated again with only NH_4_Cl and DMF, and the reaction resulted in formation of compound **13**. This experiment shows that NaN_3_/DMF does not to play any role in this transformation. The structure of **13** was elucidated with the help of the 2D NMR (COSY and HMQC) experiments. Finally, removal of the Boc group from **13** with HCl(g)–MeOH resulted in the formation of cyclic β-amino acid derivative **8** in high yield.

**Scheme 5:**
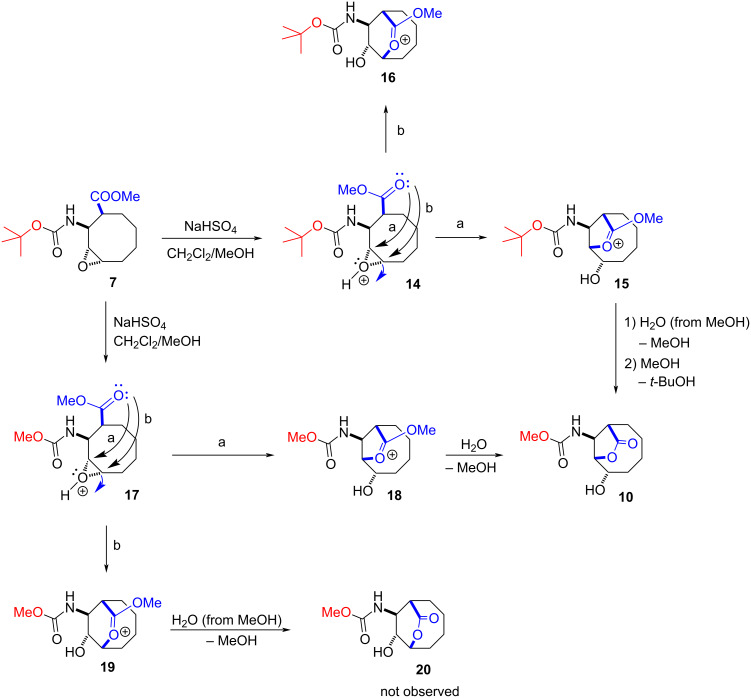
Suggested mechanism for the reaction of epoxide **7** with NaHSO_4_.

Our suggested mechanism for the reaction of epoxide **7** with NaHSO_4_ in a mixture of methylene chloride/MeOH proceeded as described in Scheme 5. First, the C=O group of the ester prefers to attack the protonated epoxide to give intermediate **15**. Then, water, which is available in methanol as an impurity, attacks the oxonium ion to give dealkylation product **10**, which is a typical transesterification reaction.

To explain the formation of lactone **10**, we performed a series of DFT computations using the Gaussian 16 software [29]. For this purpose, we performed geometry optimizations using the B3LYP functional [30–33]. Vibrational frequencies were computed to characterize each stationary structure. In all the computations, Pople’s polarized triple-ζ split valence basis set with diffuse functions, 6-311++G(d,p) [34–36], was utilized. The solvation model based on density (SMD) [37] was used to investigate the effects of methanol (ε = 32.613) and dichloromethane (ε = 8.93) on the computed Gibbs energies. For the TS between species A and B, the TSA-B notation is used throughout the article.

**Figure 3:**
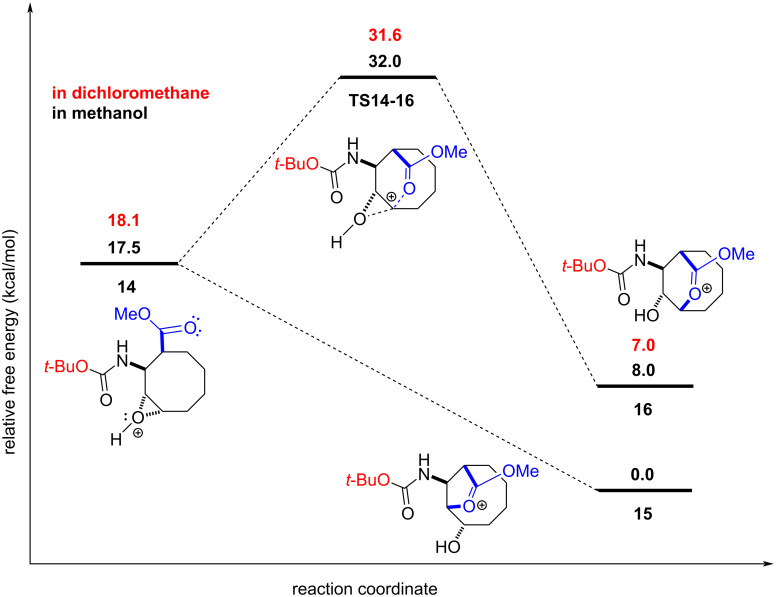
Solvent-corrected relative free energy profile at 298.15 K for the reaction mechanism of **14** shown in Scheme 5.

**Figure 4:**
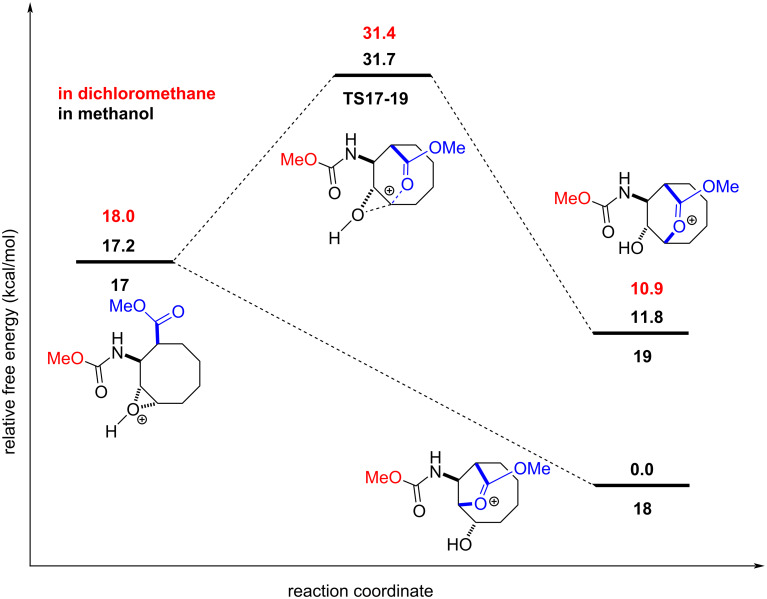
Solvent-corrected relative free energy profile at 298.15 K for the reaction mechanism of **17** shown in Scheme 5.

According to the given mechanism in Scheme 5, there are two possible paths for the attack of the carboxyl group on the epoxide ring. For the formation of the five-membered lactone **15** (path a), **14 → 15**, the reaction proceeds via a barrierless path and the solvent corrected reaction free energies are −17.5 and −18.1 kcal mol^−1^ with methanol and dichloromethane, respectively. For the formation of the six-membered lactone **16** (path b), **14 → 16**, the solvent corrected reaction free energy and barrier are −9.5 and 14.5 kcal mol^−1^ with methanol, respectively and −11.1 and 13.5 kcal mol^−1^ with dichloromethane, respectively (Figure 3). For the formation of the five-membered lactone **18** (path a), **17 → 18**, the reaction proceeds via a barrierless path and the solvent corrected reaction free energies are −17.2 and −18.0 kcal mol^−1^ with methanol and dichloromethane, respectively. For the formation of the six-membered lactone **19** (path b), **17 → 19**, the solvent corrected reaction free energy and barrier are −5.4 and 14.5 kcal mol^−1^ in methanol, respectively and −7.1 and 13.4 kcal mol^−1^ in dichloromethane, respectively (Figure 4). These results indicate that the formation of the methyl carbamate may occur before or after the attack by the carboxyl group. Overall, our computations demonstrate that the **14 → 15** and **17 → 18** conversions are kinetically more favourable, by about 14.0 kcal mol^−1^, compared to the **14 → 16** and **17 → 19** conversions. These results rule out the formations of **16** and **19** at room temperature. Therefore, we conclude that the final product is lactone **10**, and the formation of lactone **20** is not feasible under the experimental conditions. Furthermore, lactone **10** is energetically more stable, by 5.0 kcal mol^−1^ in methanol and by 3.7 kcal mol^−1^ in dichloromethane, than lactone **20**. The computations were in good agreement with the experimental results (Figure 3 and Figure 4).

**Figure 5:**
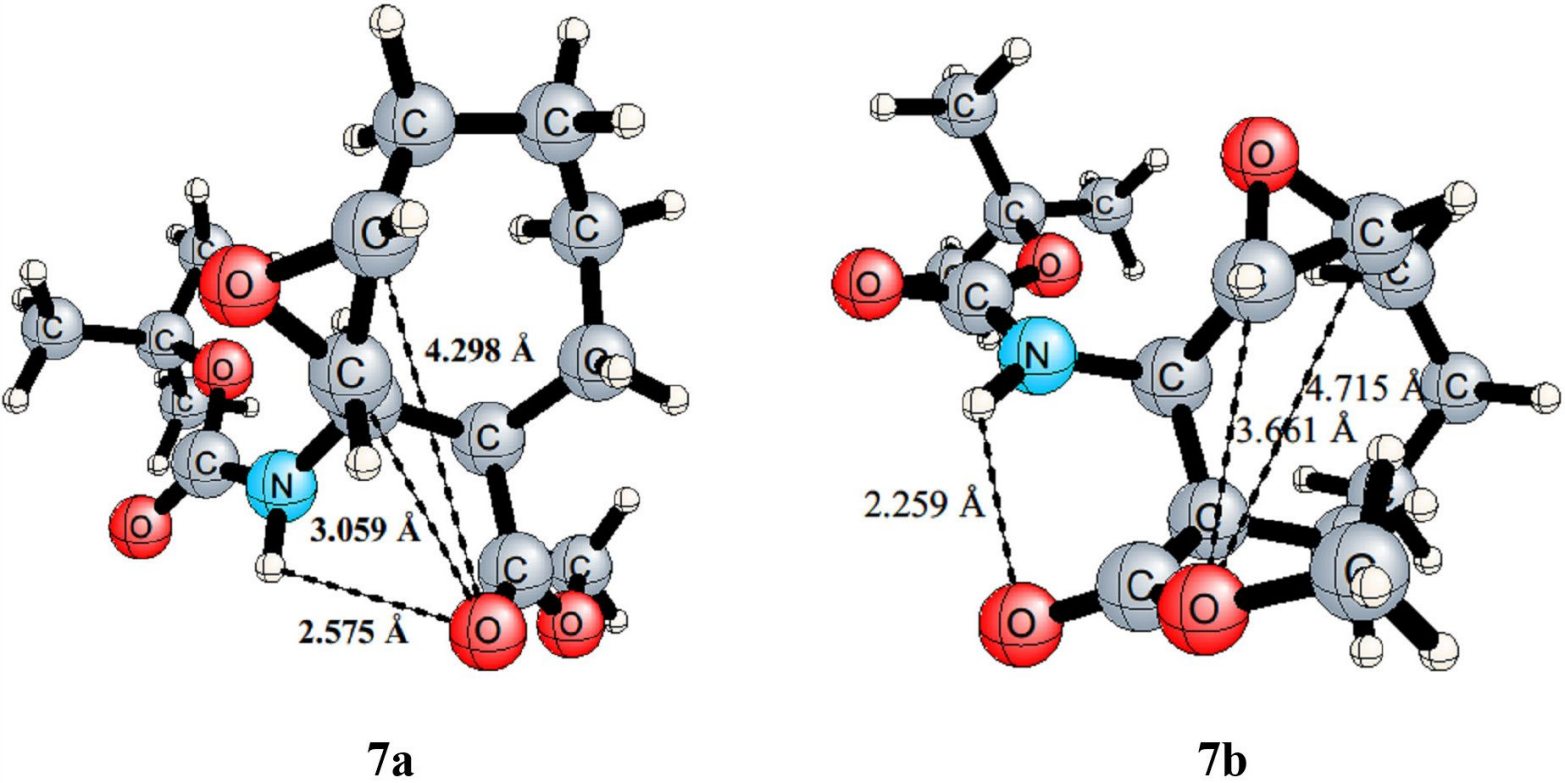
The optimized geometries of the conformers **7a** and **7b** with selected interatomic distances at the B3LYP/6-311++G(d,p) level.

To explain why lactone **10** is formed, a conformational analysis for the epoxide **7** was performed. It is seen that the conformation is quite appropriate for the formation of lactone **10**. The hydrogen bond between the C=O group of methoxy ester and NH group of the carbamate in **7** provides an appropriate conformation for the formation of lactone **10**. Additionally, according to the conformational analysis of epoxide **7**, the relative free energy of **7b** is higher by 10.3 kcal mol^−1^ than that of **7a** (Figure 5).

## Conclusion

In summary, we successfully synthesized hydroxylated cyclooctane β-amino acid **6** and its derivatives **8**, **10**, and **13** starting from β-lactam **2**. The regioselective synthesis of lactone **8**, which is a cyclic β-amino acid derivative, was achieved by oxirane ring opening in epoxide **7** with HCl(g)–MeOH, NaHSO_4_, or NH_4_Cl–DMF. The regioselectivity of oxirane ring-opening in **7** was attributed to the conformational effects. The mechanism for the formation of compound **10** was elucidated with DFT computations. Our computations demonstrate that the formation of the five-membered lactone is kinetically more favourable, and the formation of six-membered lactone is not feasible under the experimental conditions. The conformation of epoxide **7** is quite appropriate for the formation of the five-membered lactone **10**, in contrast to the formation of the six-membered lactone **20**. In addition, these novel compounds synthesized may be used as intermediates to design pharmacological tools.

## Supporting Information

Experimental section, ^1^H and ^13^C NMR spectra for all new compounds, as well as selected 2D NMR spectra and crystallographic data for compound **10** are provided. Optimized geometries of the transition states with selected interatomic distances and cartesian coordinates for computed structures are reported.

File 1 Additional experimental and computed data.
