# Supplementary material for: Efficient and regioselective synthesis of dihydroxy-substituted 2-aminocyclooctane-1-carboxylic acid and its bicyclic derivatives
Source: Beilstein J Org Chem. 2022 Jan 6;18:77–85. doi: 10.3762/bjoc.18.7 (PMC8744459; doi:10.3762/bjoc.18.7)
Supplement: File 1 — Additional experimental and computed data. [file Beilstein_J_Org_Chem-18-77-s001.pdf]

BEILSTEIN JOURNAL OF ORGANIC CHEMISTRY

# Supporting Information

for

# Efficient and regioselective synthesis of dihydroxy-substituted 2-aminocyclooctane-1-carboxylic acid and its bicyclic derivatives

İlknur Polat, Selçuk Eşsiz, Uğur Bozkaya and Emine Salamci

*Beilstein J. Org. Chem.* **2022,** *18,* 77–85. doi:10.3762/bjoc.18.7

# Additional experimental and computed data

License and Terms: This is a supporting information file under the terms of the Creative Commons Attribution License (https://creativecommons.org/licenses/by/4.0). Please note that the reuse, redistribution and reproduction in particular requires that the author(s) and source are credited and that individual graphics may be subject to special legal provisions.
The license is subject to the *Beilstein Journal of Organic Chemistry* terms and conditions: (https://www.beilstein-journals.org/bjoc/terms)

# Table of Contents

**1. Experimental section** ........ S2

**2. $^{1}H$ NMR and $^{13}C$ NMR spectra** ........ S5

**3. X-ray crystallographic data** ........ S25

**Figure S1.** X-ray crystal structure of **10** ........ S25

**Table S1.** Crystal data and structure refinement for compound **10** ........ S26

**4. Relative free energy profile** ........ S27

**Figure S2.** Relative free energy profile at 298.15 K for the reaction mechanism of **14** shown in Scheme 5. ........ S27

**Figure S3.** Relative free energy profile at 298.15 K for the reaction mechanism of **17** shown in Scheme 5. ........ S27

**5. The optimized geometries of the transition states with selected interatomic distances** ........ S28

**Figure S4.** Computed structure of **TS14-15** at the B3LYP/6-311++G(d,p) level (im. freq.= 187*i*). ........ S28

**Figure S5.** Computed structure of **TS14-16** at the B3LYP/6-311++G(d,p) level (im. freq.= 245*i*). ........ S28

**Figure S6.** Computed structure of **TS17-18** at the B3LYP/6-311++G(d,p) level (im. freq.= 193*i*). ........ S29

**Figure S7.** Computed structure of **TS17-19** at the B3LYP/6-311++G(d,p) level (im. freq.= 243*i*). ........ S29

**6. Cartesian coordinates for optimized structures** ........ S30

# 1. Experimental section

## General information

Melting points are uncorrected. Infrared spectra were obtained from solution in 0.1 mm cells or KBr pellets on an FT-IR Mattson 1000 instrument. The $^1$H and $^{13}$C NMR spectra were recorded on 400 (100) MHz Varian or 400 (100) MHz Bruker spectrometer and are reported in δ units with $SiMe_4$ as internal standard. HRMS spectra were obtained on a Bruker microTOF-Q or Agilent 6530 Accurate Mass Q-TOF instrument. Melting points were determined on a GallenKamp MPD 350 apparatus. Column chromatography was performed on silica gel (60 mesh, Merck). TLC was carried out on Merck 0.2 mm silica gel 60 $F_{254}$ analytical aluminium plates.

**(1*R**,8*S**)-9-Azabicyclo[6.2.0]dec-6-en-10-one (2):** Compound **2** was prepared as described in the literature [1].

**Methyl (1*S**,2*R**, Z)-2-aminocyclooct-3-ene-1-carboxylate (3):** To a magnetically stirred solution of β-lactam **2** (1.0 g, 6.61 mmol) in absolute methanol (10 mL) cooled to 0 °C. Then, absolute methanol (15 mL) containing 10% HCl gas was added into the reaction mixture and stirred at 0 °C for 1 h. After the mixture was stirred at room temperature for 24 h, evaporation of solvent gave **3** (1.12 g, 92%). *cis*-Amino ester **3** was recrystallized from EtOH/ether (5:5) as a white solid; mp 95-97 °C. $^1$H-NMR (400 MHz, $CD_3OD$) δ 5.88-5.64 (m, 2H, H-4 and H-3), 4.50-4.41 (m, 1H, H-2), 3.72 (bs, 3H, $OCH_3$), 3.04-2.95 (m, 1H, H-1), 2.30-1.30 (series of m, 8H, $CH_2$). $^{13}$C-NMR (100 MHz, $CD_3OD$): δ 173.9, 130.7, 128.9, 55.5, 52.0, 51.9, 29.3, 28.2, 26.5, 24.9; IR (KBr, $cm^{-1}$): 3431, 3019, 2933, 2868, 1716, 1614, 1504, 1453, 1293, 1207, 1050. HRMS (ESI) m/z: $[M + H]^+$ calcd for $C_{10}H_{17}NO_2$: 184.1338; found: 184.1332.

**Methyl (1*S**,2*R**, Z)-2-((*tert*-butoxycarbonyl)amino)cyclooct-3-ene-1-carboxylate (4):** *cis*-Amino ester **3** (1.0 g, 5.46 mmol) was dissolved in 10 mL of anhydrous pyridine and the solution was stirred under nitrogen at 0 °C. 4-(Dimethylamino)pyridine (DMAP) (10.0 mg) and $Boc_2O$ (1.23 g, 5.62 mmol) were added and the mixture was stirred at room temperature for 48 h. Then the mixture was purified by chromatography on a silica gel column eluting with EtOAc/hexane (20:80) to give pure *N*-Boc-amino ester **4** (1.47 g, 95%), as a white solid; mp 67-69 °C. $^1$H-NMR (400 MHz, $CDCl_3$) δ 5.79-5.69 (m, 1H, H-4), 5.64-5.55 (m, 1H, H-3), 5.43-5.34 (m, 1H, NH), 4.84-4.74 (m, 1H, H-2), 3.69 (s, 3H, $OCH_3$), 3.02-2.93 (m, 1H, H-1), 2.40-1.20 (series of m, 8H, $CH_2$), 1.45 (s, 9H, $CH_3$). $^{13}$C-NMR (100 MHz, $CDCl_3$) δ 174.5, 155.3, 130.2, 130.1, 79.3, 51.6, 51.0, 48.4, 29.0, 28.4, 27.8, 26.4, 24.3; IR (KBr, $cm^{-1}$): 3385, 2976, 2930, 2859, 2347, 1715, 1496, 1458, 1391, 1366, 1274, 1245, 1173. HRMS (ESI) m/z: $[M + H - C_5H_9O_2]^+$ calcd for $C_{10}H_{17}NO_2$: 184.1338; found: 184.1356. $[M + Na]^+$ calcd for $C_{15}H_{25}NO_4$: 306.1681; found: 306.1675.

**Methyl (1*S**,2*S**,3*R**,4*S**)-2-((*tert*-butoxycarbonyl)amino)-3,4-dihydroxycyclooctane-1-carboxylate (5):** To a stirred solution of *N*-Boc-amino ester **4** (1.0 g, 3.53 mmol) in 8.5 mL of acetone were added a solution of NMO (0.82 g, 7.06 mmol) in 8.5 mL of water and $OsO_4$ (ca. 17.94 mg, 0.70 mmol). The resulting mixture was stirred vigorously under nitrogen at room temperature and after 7 days, the reaction was stopped. Sodium hydrosulfite (0.50 g) and silica gel (1.0 g) slurried in water (5 mL) were added, stirred for 30 min, and then filtered through a pad of Celite (1.0 g) in a 50 mL sintered glass funnel. The Celite cake was washed with acetone (3x30 mL). After evaporation of the solvents, the reaction mixture was extracted with ethyl acetate (4x30 mL). The organic solution was dried over $Na_2SO_4$. Evaporation of solvent gave pure diol **5** (1.02 g, 91%) as the sole product; a light brown solid; mp 101-103 °C. $^1$H-NMR (400 MHz, $CDCl_3$) δ 5.39 (bd, *J*= 8.9 Hz, 1H, NH), 4.19-4.05 (m, 1H, H-2), 3.99-3.90 (m, 2H, H-3 and H-4), 3.70 (s, 3H, $OCH_3$), 3.31-3.15 (m, 2H, OH), 3.04-2.96 (m, 1H, H-1), 2.11-1.20 (series of m, 8H, $CH_2$), 1.40 (s, 9H, $CH_3$). $^{13}$C NMR (100 MHz, $CDCl_3$) δ 174.6, 156.9, 80.1, 72.3, 72.1, 52.0, 50.4,

46.3, 32.0, 28.3, 26.7, 25.1, 23.9; IR (KBr, $cm^{-1}$): 3397, 2931, 2358, 2341, 1718, 1524, 1366, 1251, 1171, 1012, 737. HRMS (ESI) m/z: $[M + H]^+$ calcd for $C_{15}H_{27}NO_6$: 318.1917; found: 318.1911.

**(1*S**,2*S**,3*R**,4*S**)-2-Amino-3,4-dihydroxycyclooctane-1-carboxylic acid (6):** Ester diol **5** (500 mg, 1.58 mmol) was dissolved in 10 mL of 6 M HCl solution. The reaction mixture was refluxed for 7 h and then the solvent was removed under reduced pressure. *β*-Amino acid **6** was obtained as a viscous and yellow oil (288 mg, 90%). $^1$H NMR (400 MHz, $D_2O$) δ 3.98 (dd, *J*= 9.3, 2.2 Hz, 1H, H-2), 3.89 (ddd, *J*= 7.1, 4.7, 2.2 Hz, 1H, H-4), 3.59 (dd, *J*= 9.4, 3.0 Hz, 1H, H-3), 3.08 (ddd, *J*= 8.4, 5.0, 3.3 Hz, 1H, H-1) 1.96-1.33 (series of m, 8H, $CH_2$). $^{13}$C NMR (100 MHz, $D_2O$) δ 176.3, 71.8, 70.9, 52.6, 42.4, 30.8, 25.8, 24.3, 22.5; IR (KBr, $cm^{-1}$): 3302, 2934, 1711, 1595, 1473, 1408, 1207, 1115, 1048, 817, 610. HRMS (ESI) m/z: $[M + H]^+$ calcd for $C_9H_{17}NO_4$: 204.1236; found: 204.1234.

**Methyl (1*R**,2*S**,3*S**,8*S**)-2-((*tert*-butoxycarbonyl)amino)-9-oxabicyclo[6.1.0]nonane-3-carboxylate (7):** In a similar manner as described in the literature [2], *m*-CPBA (77 %, 0.79 g, 3.53 mmol) was added to a stirred solution of *N*-Boc-amino ester **4** (1.0 g, 3.53 mmol) in $CH_2Cl_2$ (50 mL) and $NaHCO_3$ (0.32 g, 3.81 mmol) at room temperature. The resulting mixture was stirred 6 days at room temperature, and then cooled to 0 °C. To the mixture, saturated $NaHCO_3$ solution (30 mL) was added and stirred for 10 min. The mixture was extracted with $CH_2Cl_2$ (4x25 mL). The combined organic extracts were washed with saturated NaCl solution (10 mL) and then dried ($Na_2SO_4$). Evaporation of solvent gave **7** (0.99 g, 94%) as the sole product. The epoxide **7** was recrystallized from $CH_2Cl_2$/hexane (8:2) as a colourless needle; mp 102-103 °C. $^1$H NMR (400 MHz, $CDCl_3$) δ 5.31 (bd, *J*= 8.5 Hz, NH, 1H), 3.80-3.62 (m, 1H, H-2), 3.69 (s, 3H, $OCH_3$), 3.39 (dd, *J*= 9.5, 4.4 Hz, 1H, H-3), 3.04-2.92 (m, 2H, H-1 and H-8), 2.35-1.20 (series of m, 8H, $CH_2$), 1.41 (s, 9H, $CH_3$). $^{13}$C NMR (100 MHz, $CDCl_3$) δ 174.7, 155.6, 79.9, 56.0, 55.6, 52.0, 50.8, 49.2, 28.6, 28.3, 27.7, 25.2, 24.6; IR (KBr, $cm^{-1}$): 3371, 2974, 2933, 2870, 2347, 1713, 1501, 1460, 1366, 1171. HRMS (ESI) m/z: $[M + H – C_5H_9O_2]^+$ calcd for $C_{10}H_{17}NO_3$: 200.1287; found: 200.1281.

**Reaction of epoxide 7 with HCl(g) in MeOH:** Epoxide **7** (1.0 g, 3.34 mmol) was hydrolysed with HCl(g) in MeOH (10%) as described above for the synthesis of **3** to obtain product of the ring-opening. Evaporation of the solvent gave a mixture of products **8** and **9**. From $^1$H NMR spectroscopy was observed that the mixture of **8** and **9** in a 9:1 ratio. The product **8** in the reaction mixture was purified by recrystallization from EtOH−ether (5:5) as a white solid (493 mg, 80%), mp 229-231 °C; but all attempts to purify the expected product **9** (28 mg, 4%) failed.
(1*S**,6*S**,7*S**,10*S**)-10-Amino-6-hydroxy-8-oxabicyclo[5.2.1]decan-9-one (**8**): $^1$H NMR (400 MHz, $CD_3OD$) δ 4.89 (bs, $NH_2$/OH), 4.62 (dd, $J_{6,7}$= 6.5 Hz, $J_{7,10}$ =1.3 Hz, 1H, H-7), 4.39 (t, *J*= 1.6 Hz, 1H, H-10), 4.26 (ddd, $J_{6,7}$= 6.5 Hz, *J*= 1.9 Hz, *J*= 1.3 Hz, 1H, H-6), 2.94 (dt, *J*= 6.2, 2.0 Hz, 1H, H-1), 2.29-1.25 (series of m, 8H, $CH_2$). $^{13}$C NMR (100 MHz, $CD_3OD$) δ 176.4, 81.5, 66.5, 51.6, 44.0, 33.0, 30.5, 27.3, 17.3; IR (KBr, $cm^{-1}$): 3329, 2929, 2852, 1777, 1602, 1451, 1184, 1014, 919. HRMS (ESI) m/z: $[M + H]^+$ calcd for $C_9H_{15}NO_3$: 186.1130; found: 186.1125.

**Reaction of epoxide 7 with $NaHSO_4$ in methylene chloride/MeOH:** To a magnetically stirred solution of epoxide **7** (500 mg, 1.67 mmol) in 10 mL of methylene chloride/MeOH (1:1) was added $NaHSO_4$ (401 mg, 3.34 mmol). The mixture was stirred at room temperature for 7 days. The residue was removed by filtration. Evaporation of the solvent gave a mixture of **10** and **11** in a 7:3 ratio (from $^1$H NMR). The mixture was purified by chromatography on a chromatotron plate (4 mm, silica gel) eluted with EtOAc/hexane (1:1). Purification gave as the first fraction carbamate **10** (264 mg, 65%) and as the second diol isomer mixture **11** (130 mg, 25%). But this mixture **11** could not be obtained in pure form, although all chromatographic purification methods. The carbamate **10** was recrystallized from methylene chloride/acetone (9:1) as a colourless crystal; mp 139-140 °C.

**Methyl ((1*S**,6*S**,7*S**,10*S**)-6-hydroxy-9-oxo-8-oxabicyclo[5.2.1]decan-10-yl)carbamate (10):** $^1$H NMR (400 MHz, acetone-$d_6$) δ 7.0 (bs, NH, 1H), 4.58-4.52 (m, 1H, H-10), 4.49 (dd, *J*= 6.4, 1.6 Hz, 1H, H-7), 4.44-4.38 (m, OH, 1H), 4.23-

4.15 (m, 1H, H-6), 3.62 (s, 3H, $OCH_3$), 2.75-2.67 (m, 1H, H-1), 2.20-1.20 (series of m, 8H, $CH_2$). $^{13}C$ NMR (100 MHz, acetone-$d_6$) δ 177.4, 156.9, 84.6, 67.6, 52.5, 51.5, 47.1, 33.7, 31.4, 28.1, 18.2; IR (KBr, $cm^{-1}$): 3417, 3358, 2932, 2621, 1767, 1697, 1533, 1188, 1041, 1020. HRMS (ESI) m/z: $[M + H]^+$ calcd for $C_{11}H_{17}NO_5$: 244.1185; found: 244.1180.

**Reaction of epoxide 7 with $NaN_3$, and synthesis of *tert*-butyl ((1*S**,6*S**,7*S**,10*S**)-6-hydroxy-9-oxo-8-oxabicyclo[5.2.1]decan-10-yl)carbamate (13):** Epoxide **7** (0.50 g, 1.67 mmol) was dissolved in 10 mL of DMF in a pressure tube. To the mixture was added $NaN_3$ (0.65 g, 10.00 mmol) and $NH_4Cl$ (0.17 g, 3.18 mmol). The reaction mixture was refluxed for 4 days. After the mixture was hydrolysed by adding 8 ml of water, the solution was extracted with ethyl acetate (4x30 mL). The combined organic extracts were washed with water (4x10 mL) and then dried ($Na_2SO_4$). Evaporation of solvent gave **13** (0.38 g, 80%) as the sole product, a white solid; mp 184-185 °C. $^1H$ NMR (400 MHz, $CDCl_3$) δ 5.19 (bd, *J*= 4.5 Hz, NH, 1H), 4.58 (bd, *J*= 6.1 Hz, 1H, H-7), 4.51-4.45 (m, 1H, H-10), 4.33-4.26 (m, 1H, H-6), 3.99 (bs, 1H, OH), 2.68-2.61 (m, 1H, H-1), 2.32-1.20 (series of m, 8H, $CH_2$), 1.46 (s, 9H, $CH_3$). $^{13}C$ NMR (100 MHz, $CDCl_3$) δ 177.4, 156.1, 86.0, 81.2, 68.2, 52.1, 45.7, 33.0, 31.6, 29.7, 28.3, 27.7, 17.9; IR (KBr, $cm^{-1}$): 3423, 3417, 2978, 2930, 2621, 1771, 1685, 1185, 1020. HRMS (ESI) m/z: $[M + Na]^+$ calcd for $C_{14}H_{23}NO_5$: 308.1474; found: 308.1474.

**(1*S**,6*S**,7*S**,10*S**)-10-Amino-6-hydroxy-8-oxabicyclo[5.2.1]decan-9-one (8):** Epoxide **7** (500 mg, 1.75 mmol) was hydrolysed with HCl(g) in MeOH (10%) as described above for the synthesis of carbamate **13** to give lactone **8** (276 mg, 85%), as a white solid. Data of compound **8** were described above in the reaction of epoxide **7** with HCl(g) in MeOH.

## 2. $^{1}$H NMR and $^{13}$C NMR spectra

**Methyl (1*S**,2*R**, Z)-2-aminocyclooct-3-ene-1-carboxylate (3): $CD_3OD$ ($^{1}$H NMR and $^{13}$C NMR)**

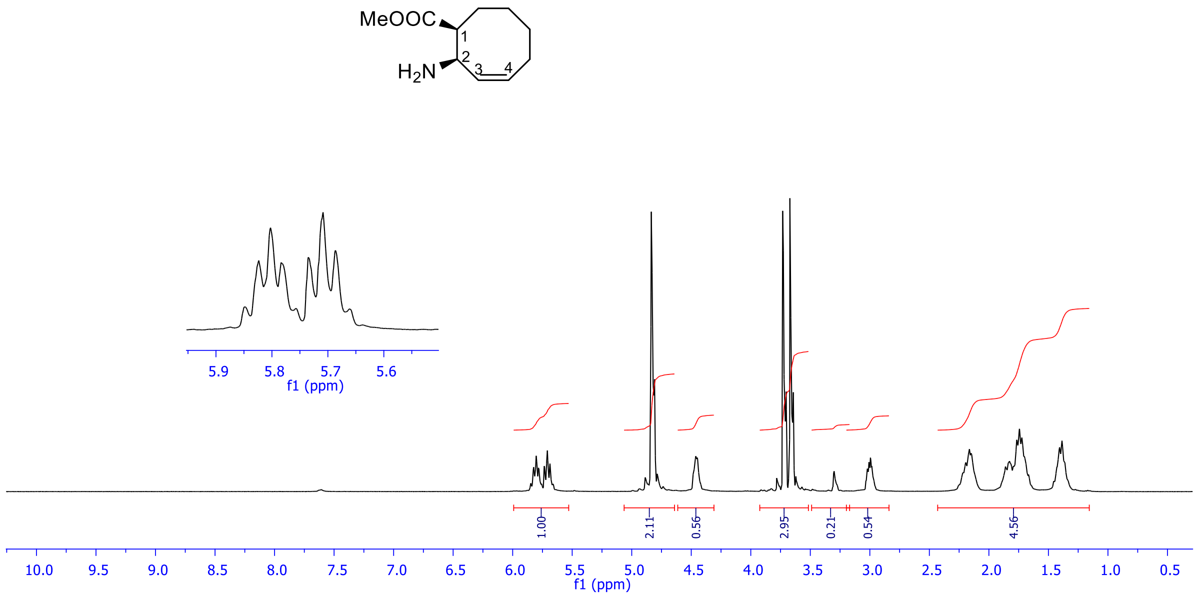


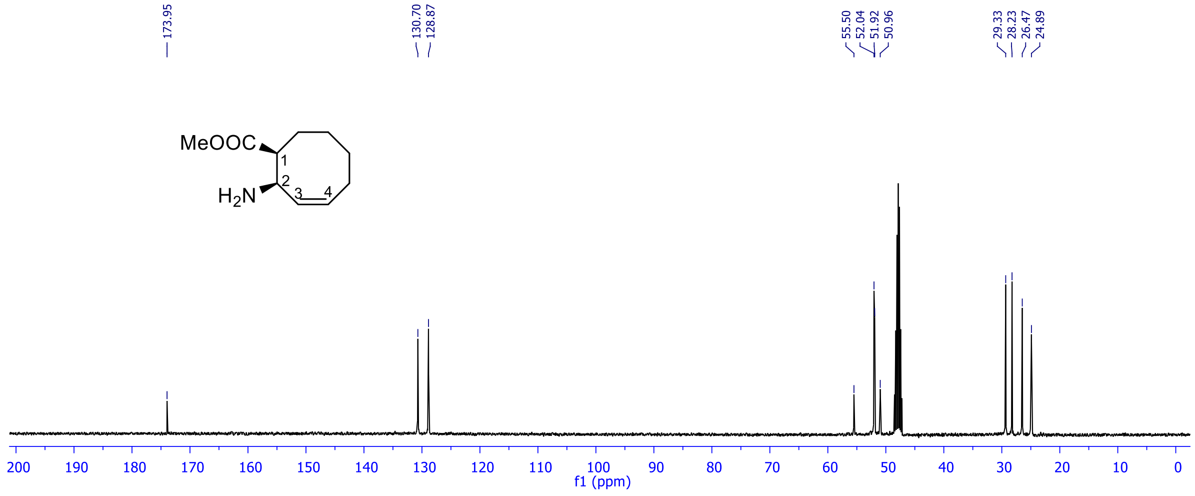

**Methyl (1*S**,2*R**, Z)-2-((*tert*-butoxycarbonyl)amino)cyclooct-3-ene-1-carboxylate (4): $CDCl_3$ ($^1$H NMR and $^{13}$C NMR)**

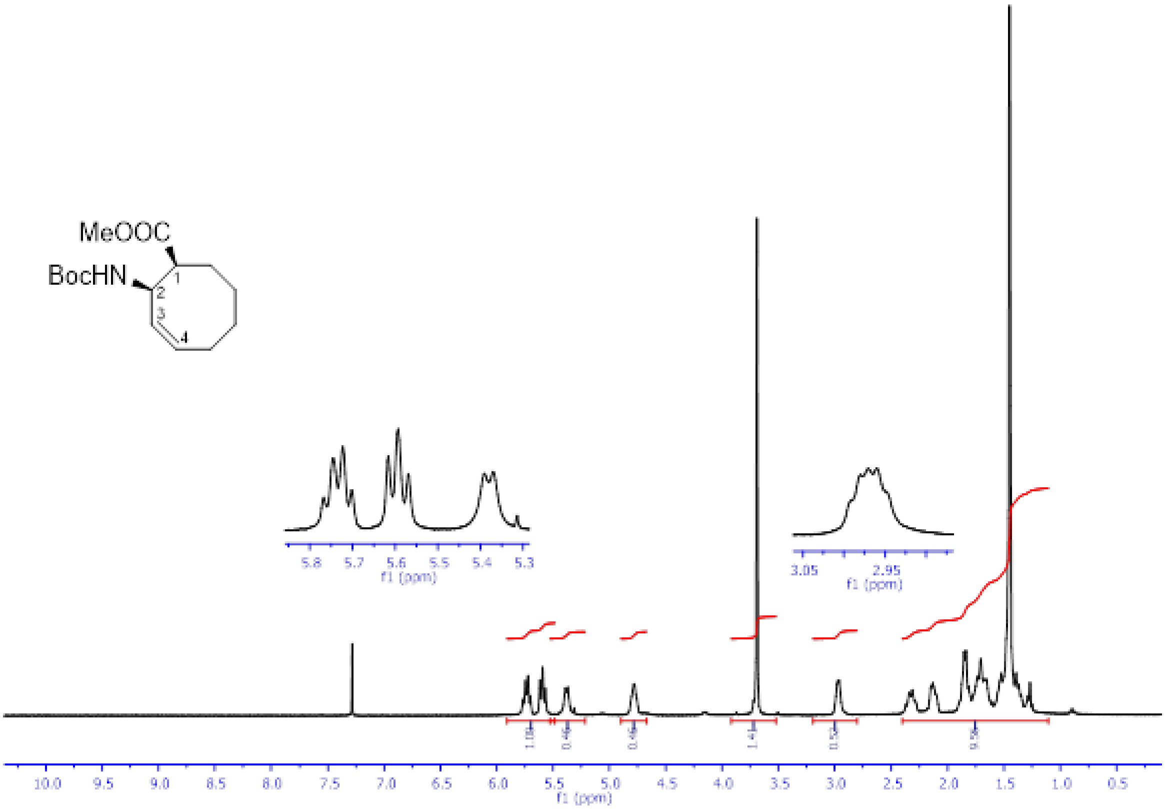


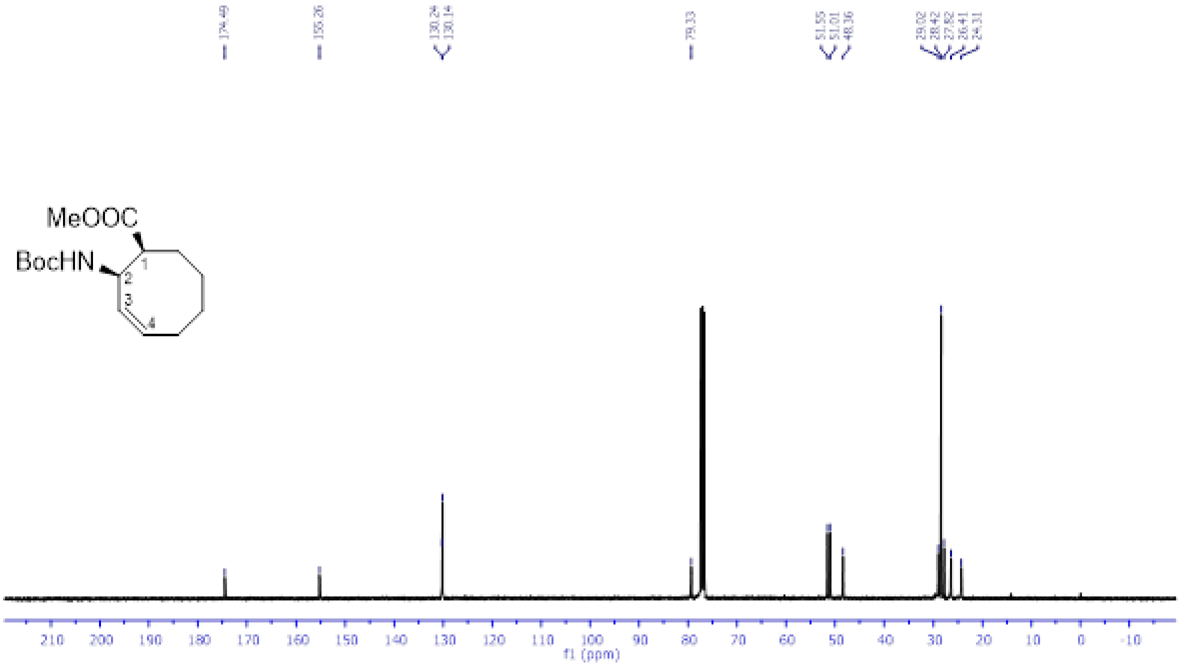

**Methyl (1*S**,2*R**, Z)-2-((*tert*-butoxycarbonyl)amino)cyclooct-3-ene-1-carboxylate (4): $CDCl_3$ (HMQC)**

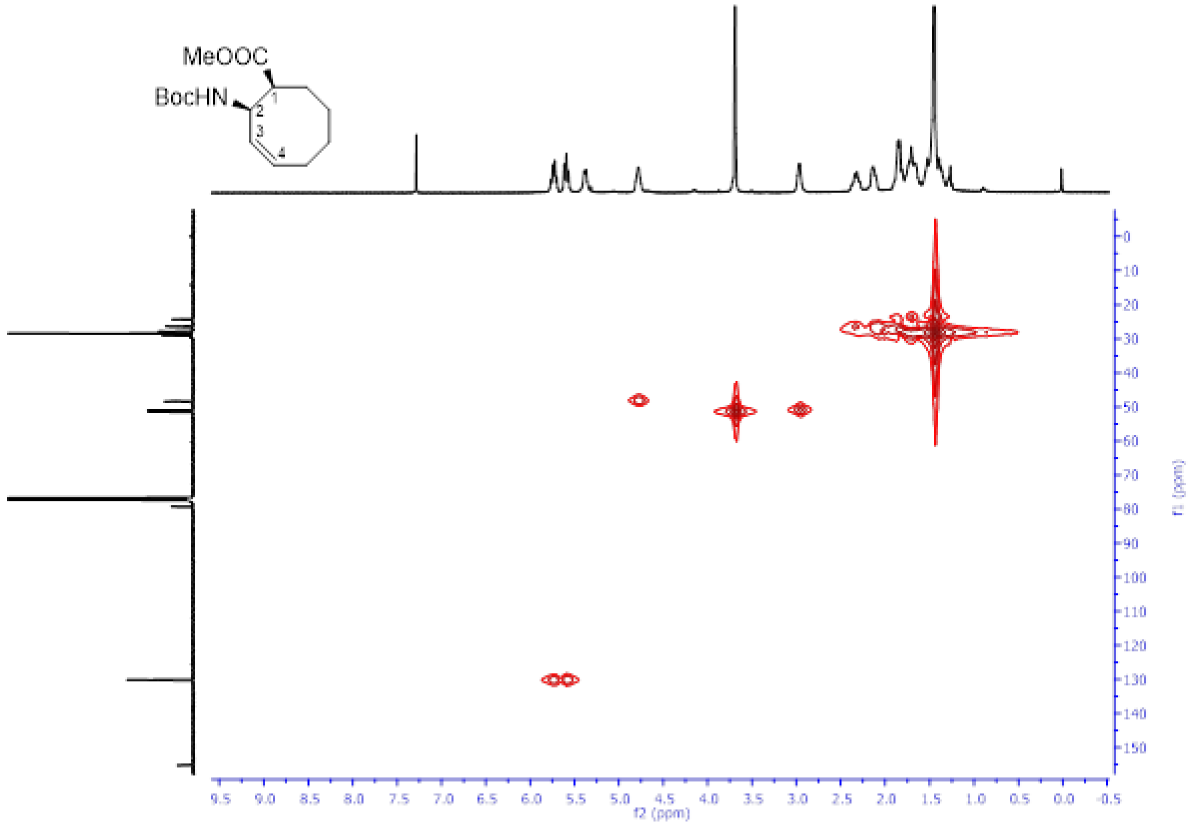


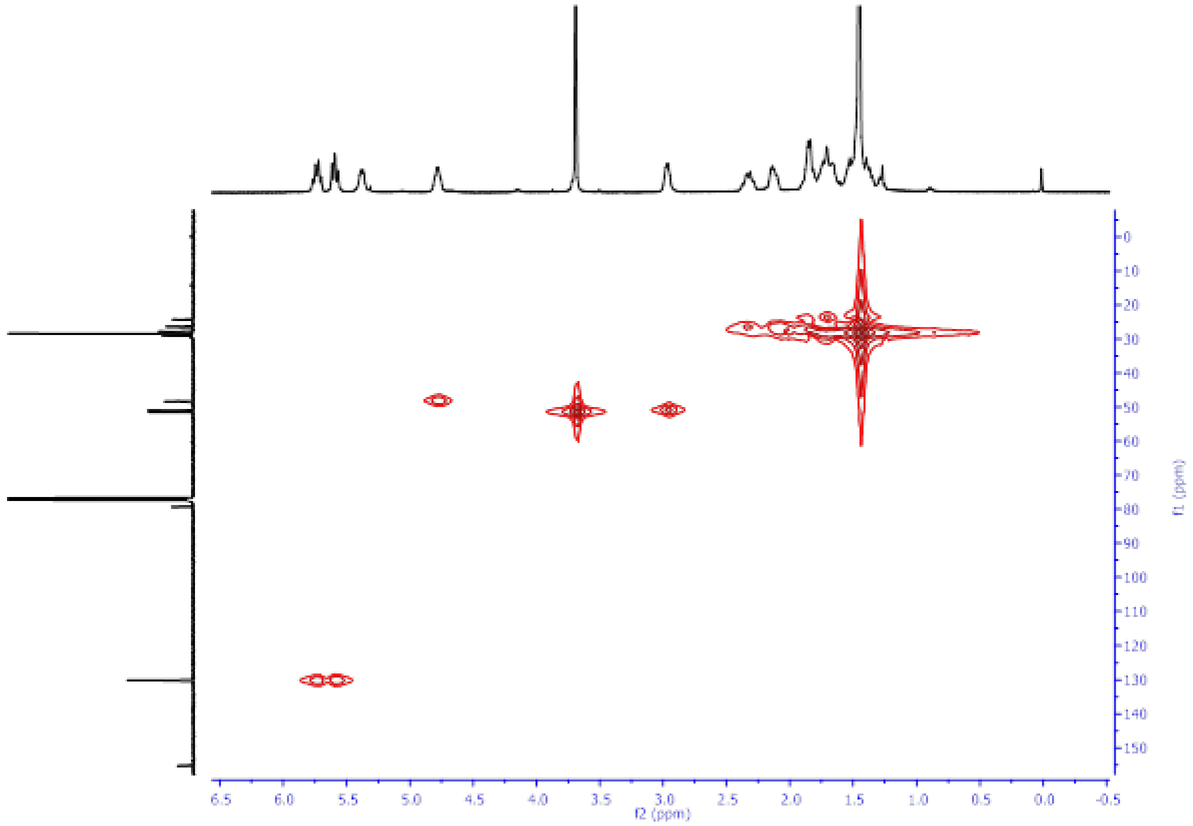

**Methyl (1*S**,2*R**, Z)-2-((*tert*-butoxycarbonyl)amino)cyclooct-3-ene-1-carboxylate (4): $CDCl_3$ (COSY)**

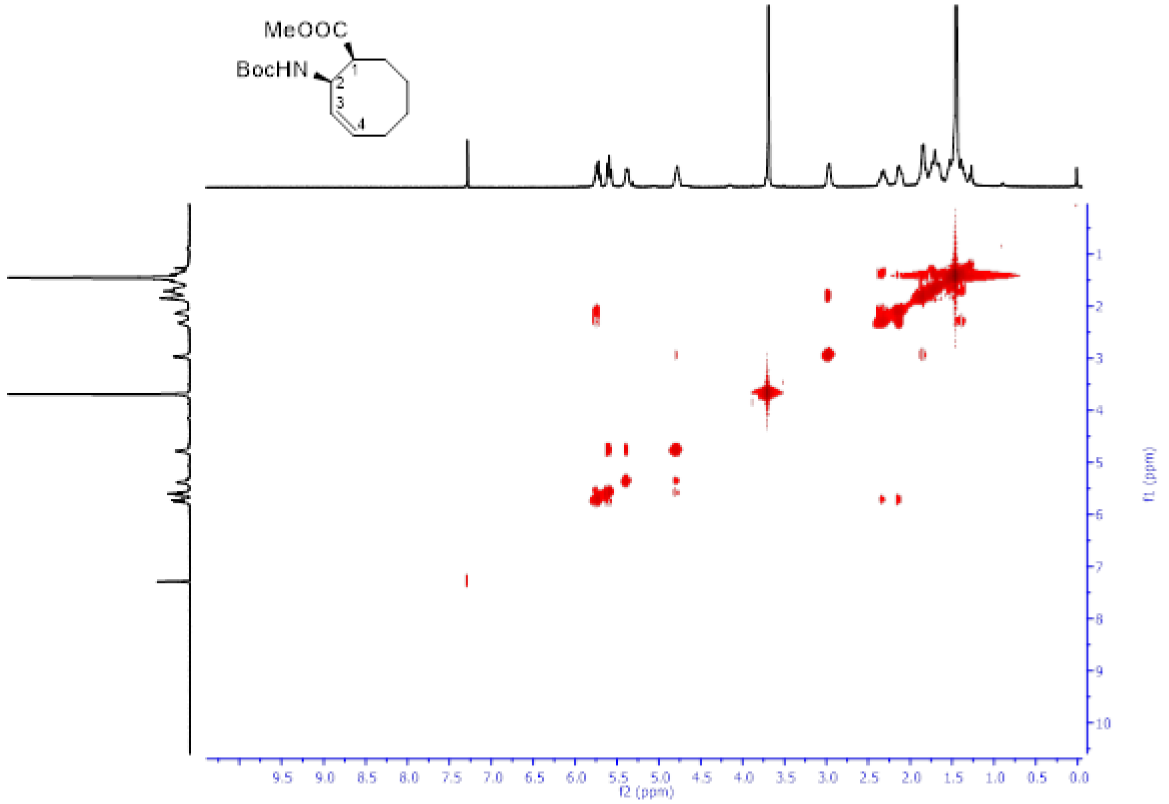


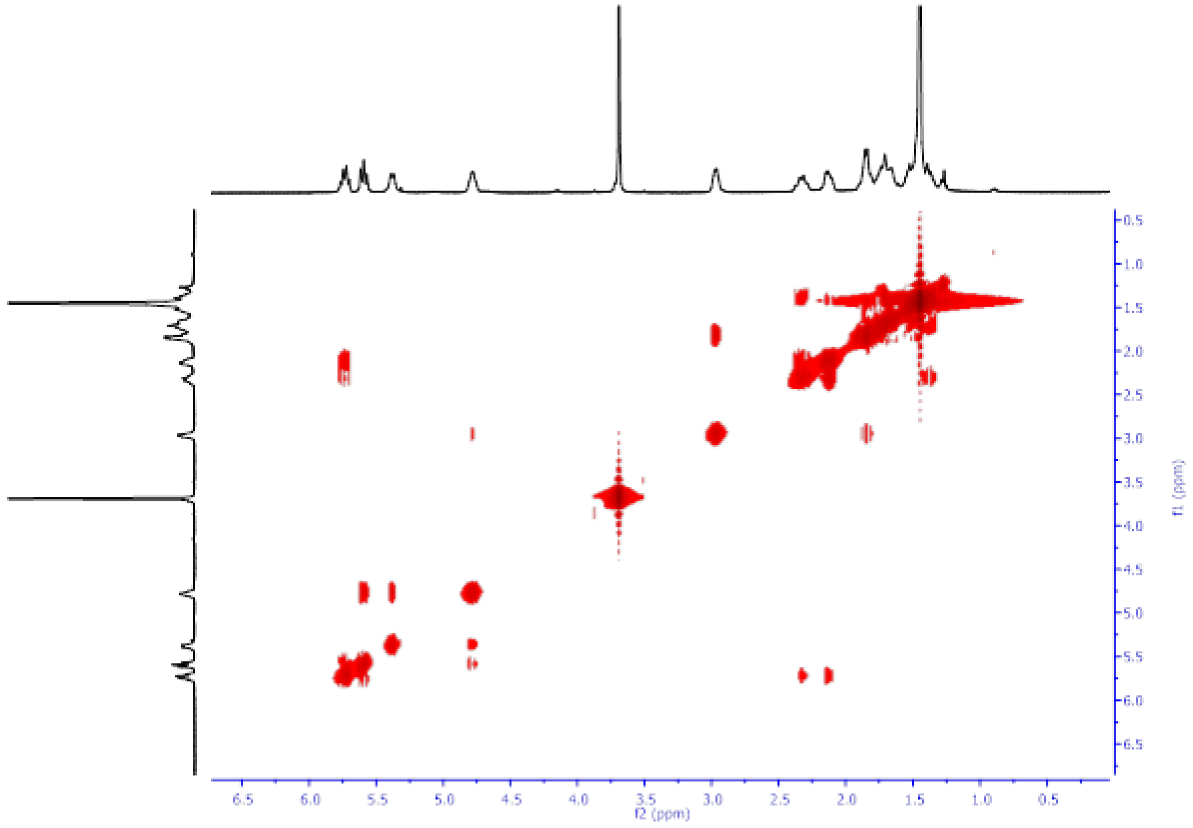

**Methyl (1*S**,2*S**,3*R**,4*S**)-2-((*tert*-butoxycarbonyl)amino)-3,4-dihydroxycyclooctane-1-carboxylate (5): $CDCl_3$ ($^1H$ NMR and $^{13}C$ NMR)**

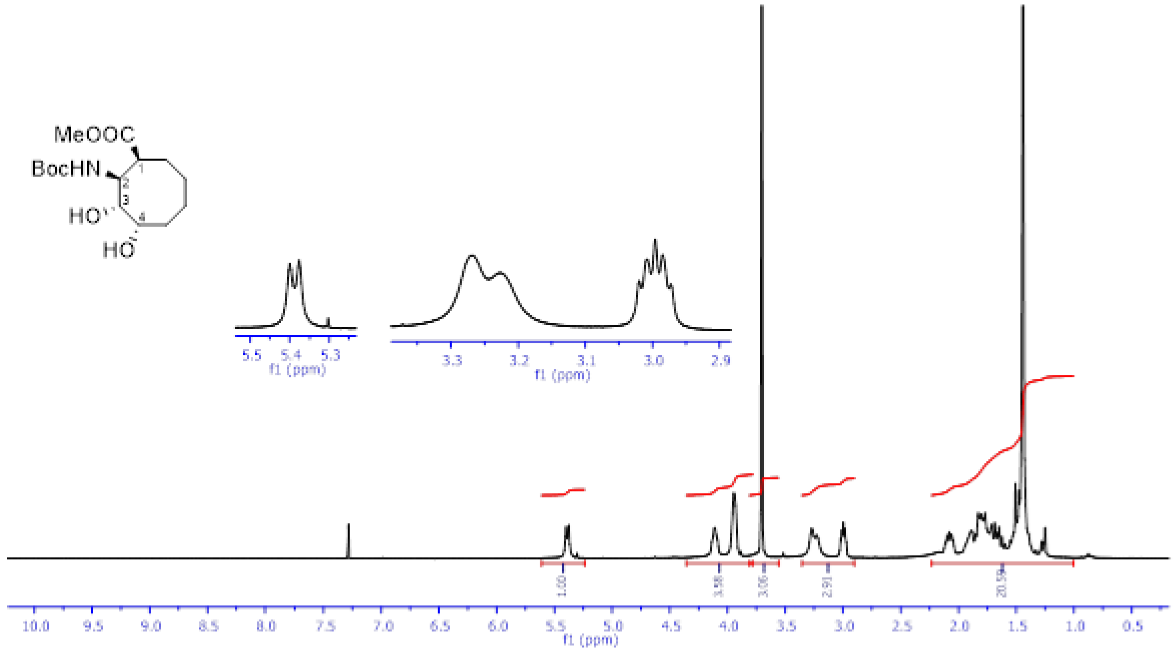

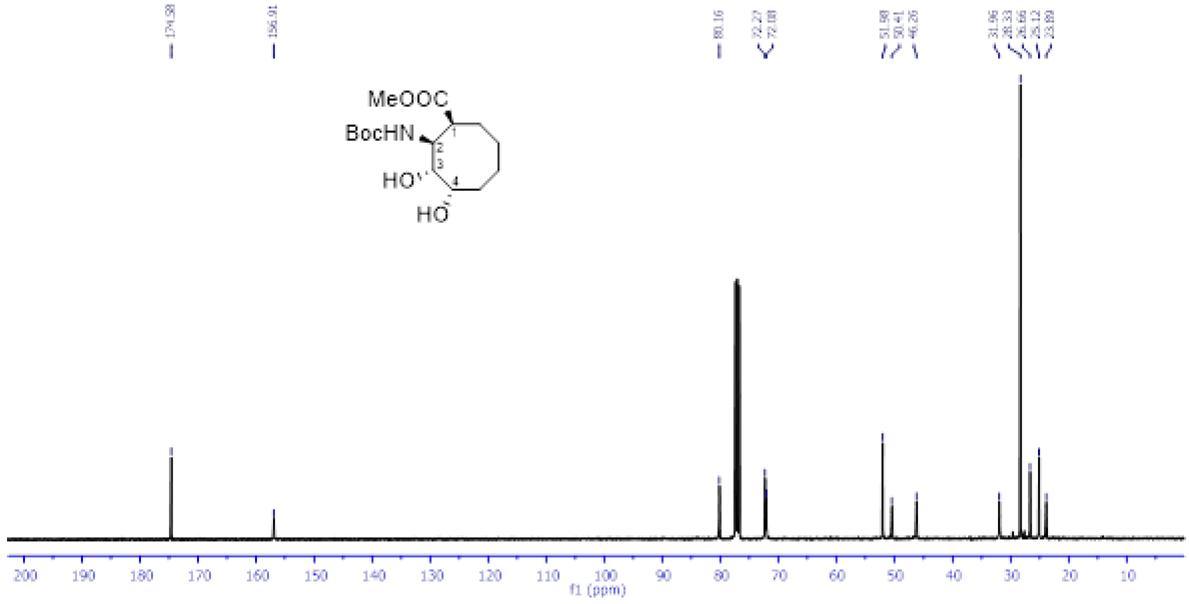

**Methyl (1*S**,2*S**,3*R**,4*S**)-2-((*tert*-butoxycarbonyl)amino)-3,4-dihydroxycyclooctane-1-carboxylate (5): $CDCl_3$ (HMQC)**

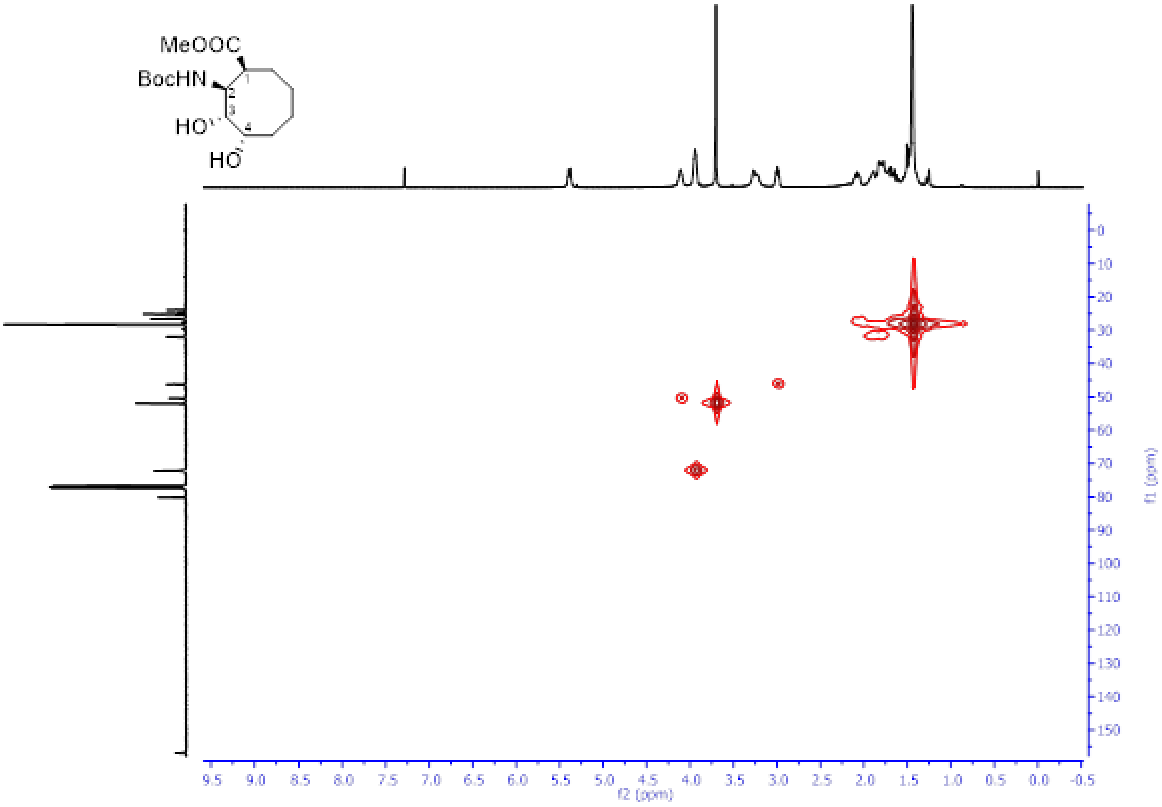


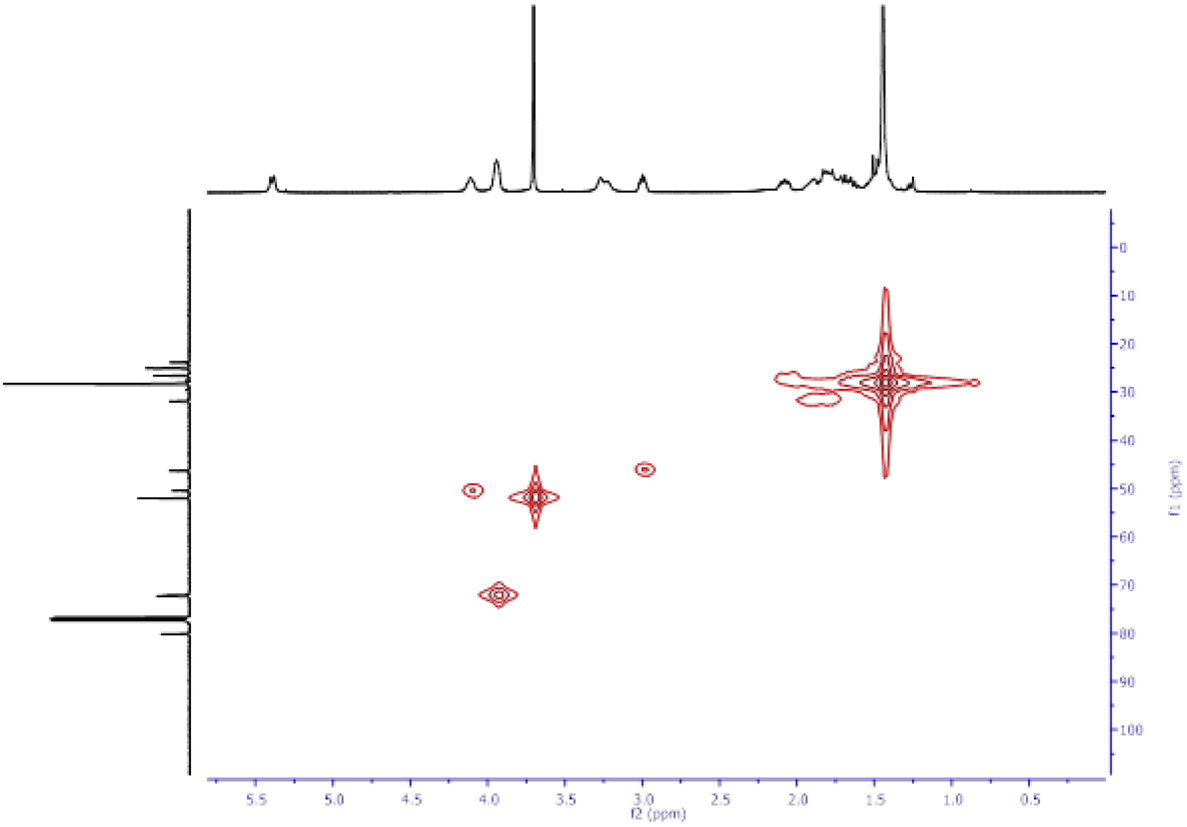

**Methyl (1*S**,2*S**,3*R**,4*S**)-2-((*tert*-butoxycarbonyl)amino)-3,4-dihydroxycyclooctane-1-carboxylate (5): CDCl$_3$ (COSY)**

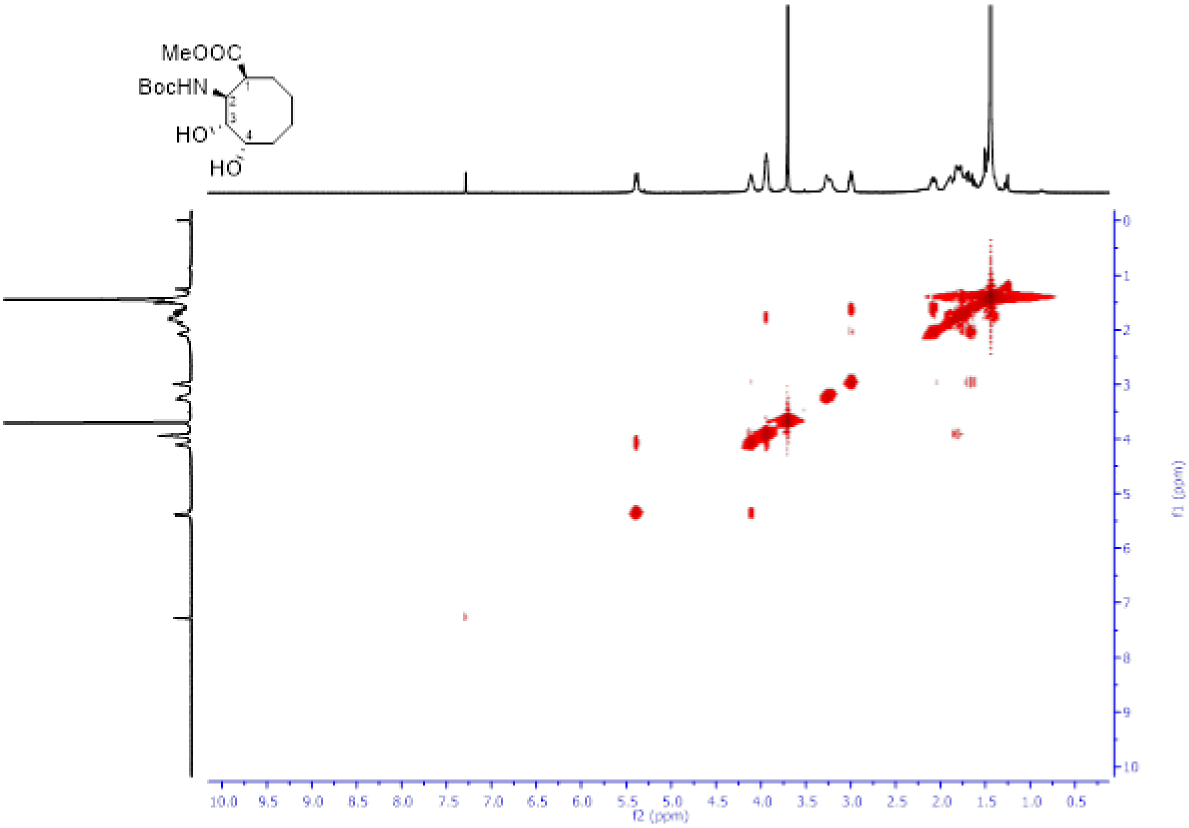


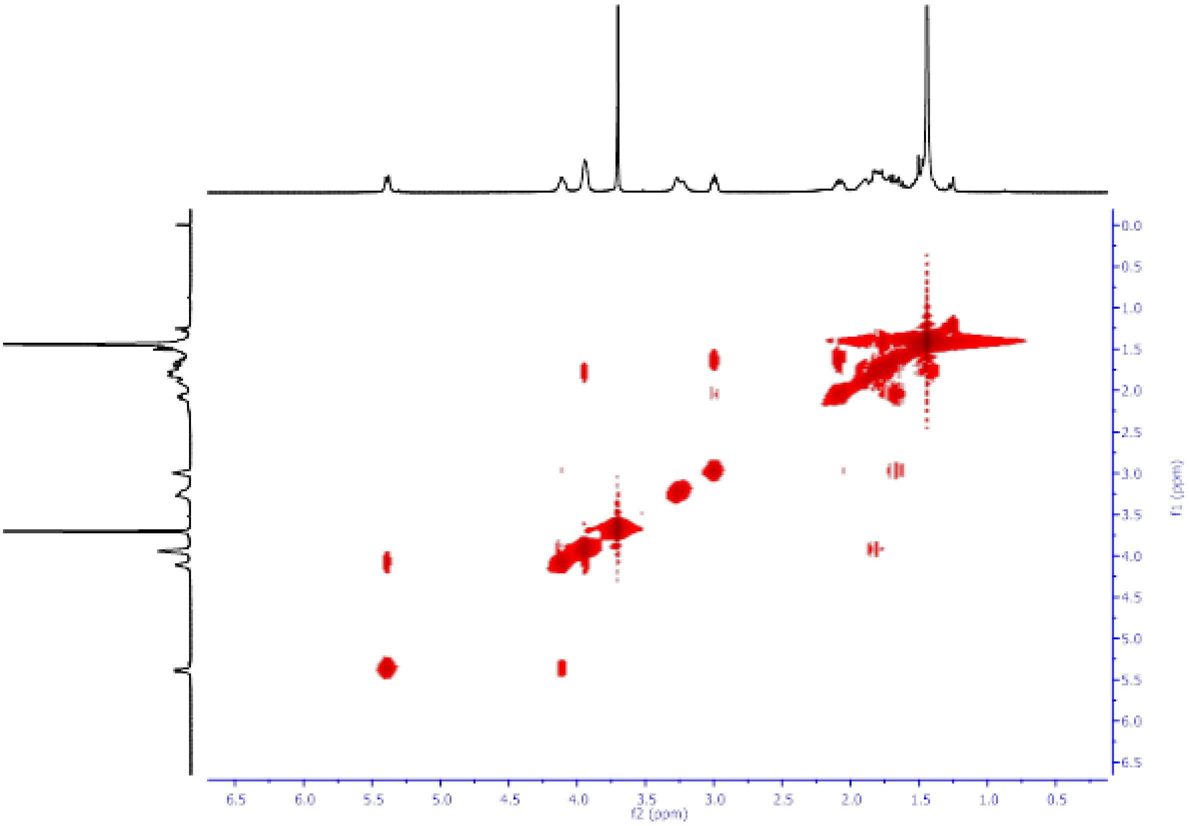

**(1*S**,2*S**,3*R**,4*S**)-2-Amino-3,4-dihydroxycyclooctane-1-carboxylic acid (6): $D_2O$ ($^1H$ NMR and $^{13}C$ NMR)**

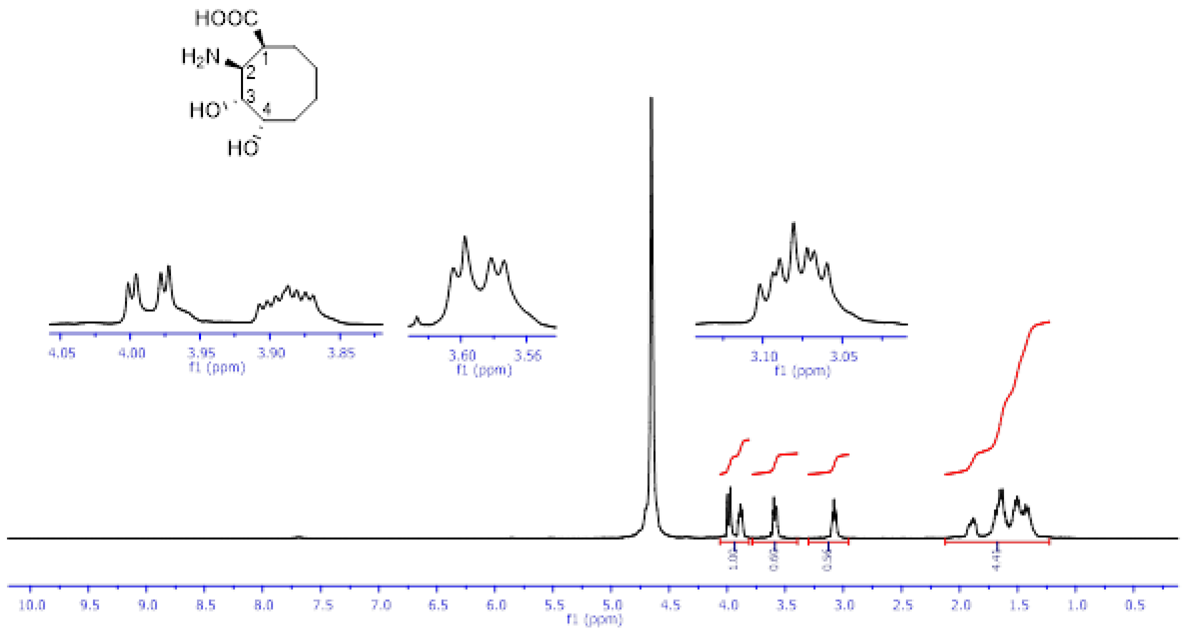


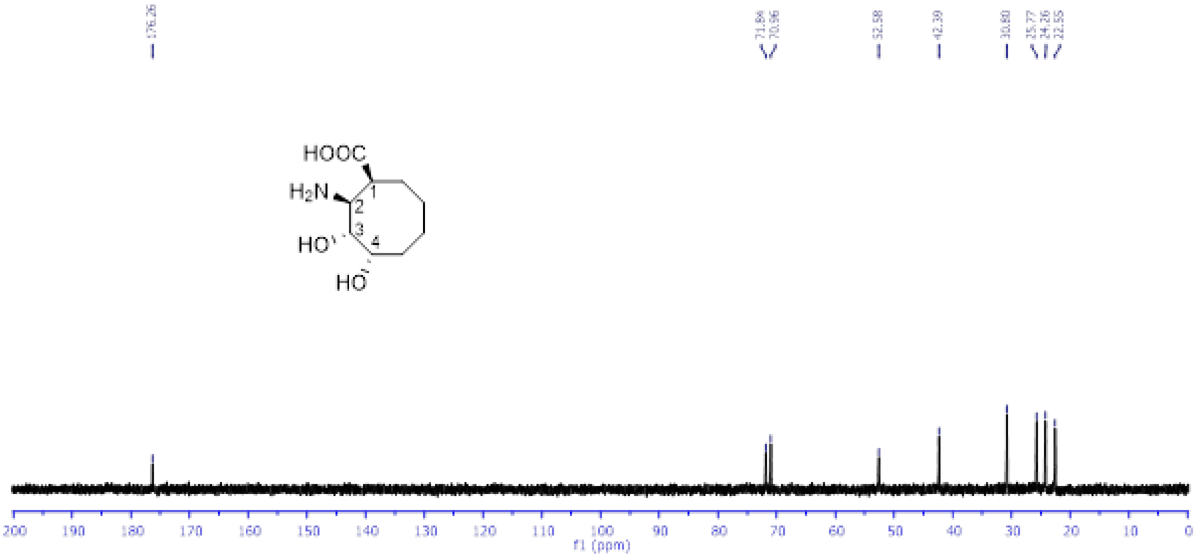

**Methyl (1*R**,2*S**,3*S**,8*S**)-2-((*tert*-butoxycarbonyl)amino)-9-oxabicyclo[6.1.0]nonane-3-carboxylate (7): CDCl3 ($^{1}$H NMR and $^{13}$C NMR)**

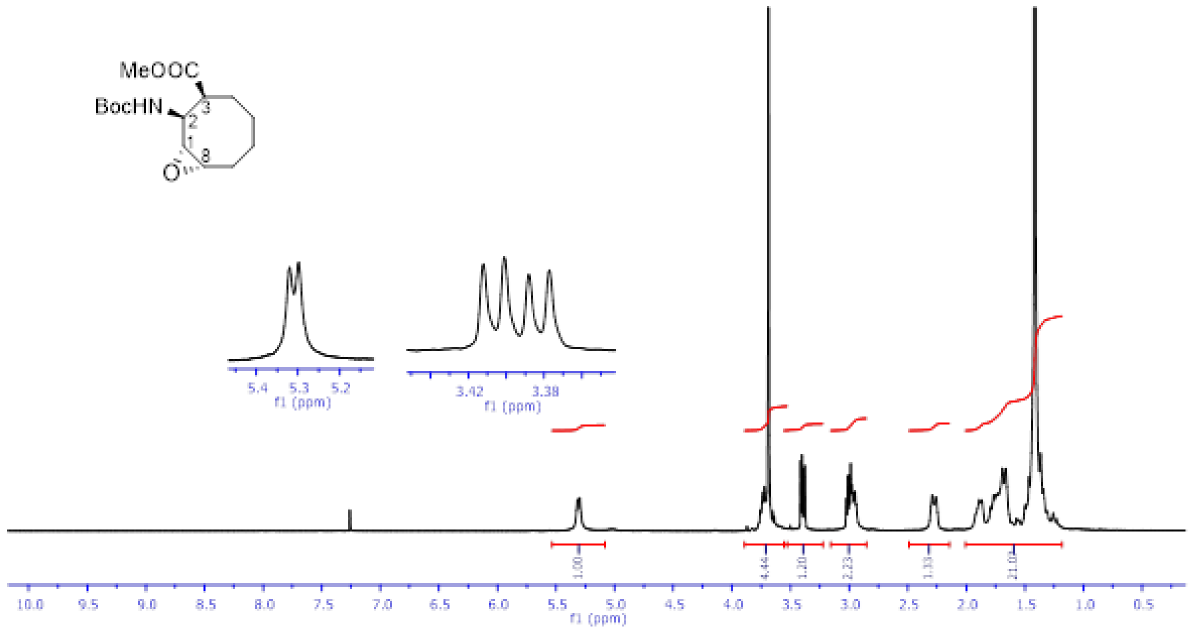

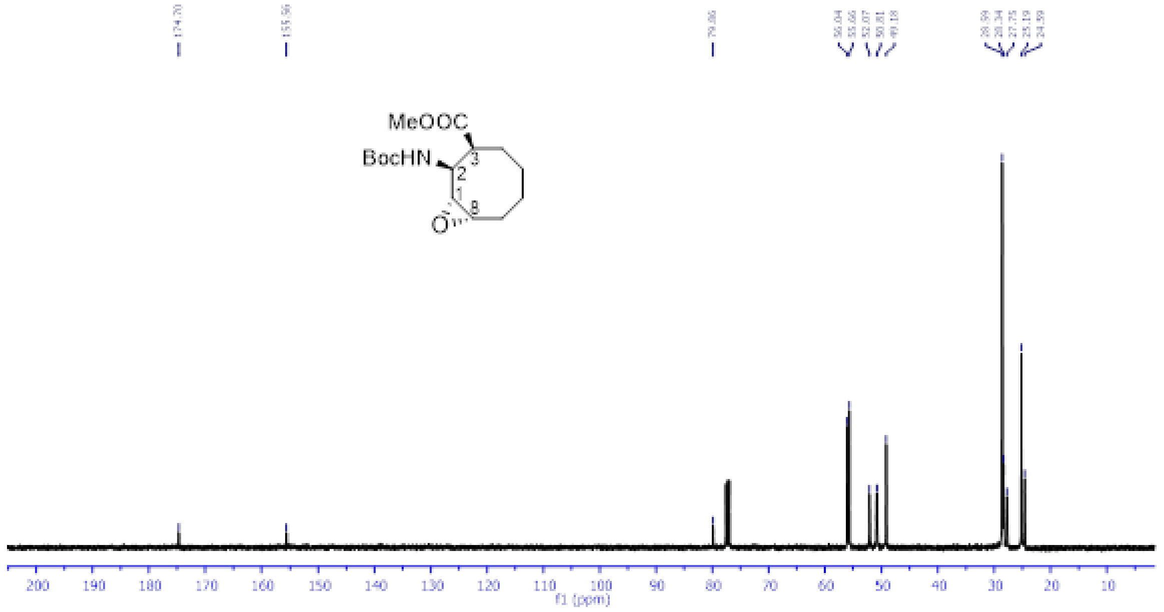

**(1*S**,6*S**,7*S**,10*S**)-10-Amino-6-hydroxy-8-oxabicyclo[5.2.1]decan-9-one (8): $CD_3OD$ ($^1$H NMR and $^{13}$C NMR)**

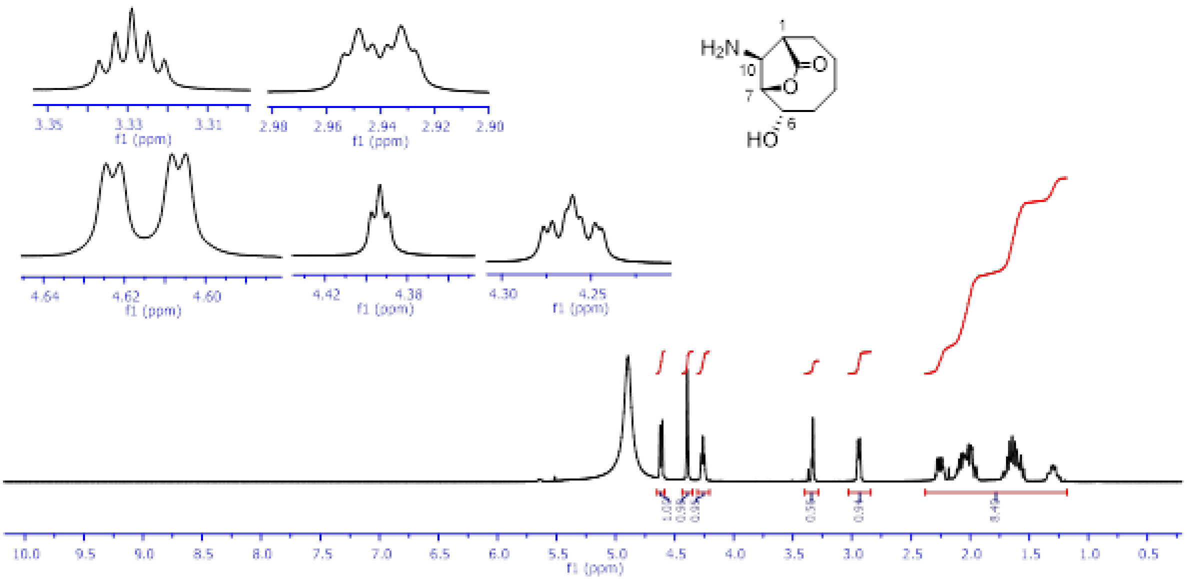


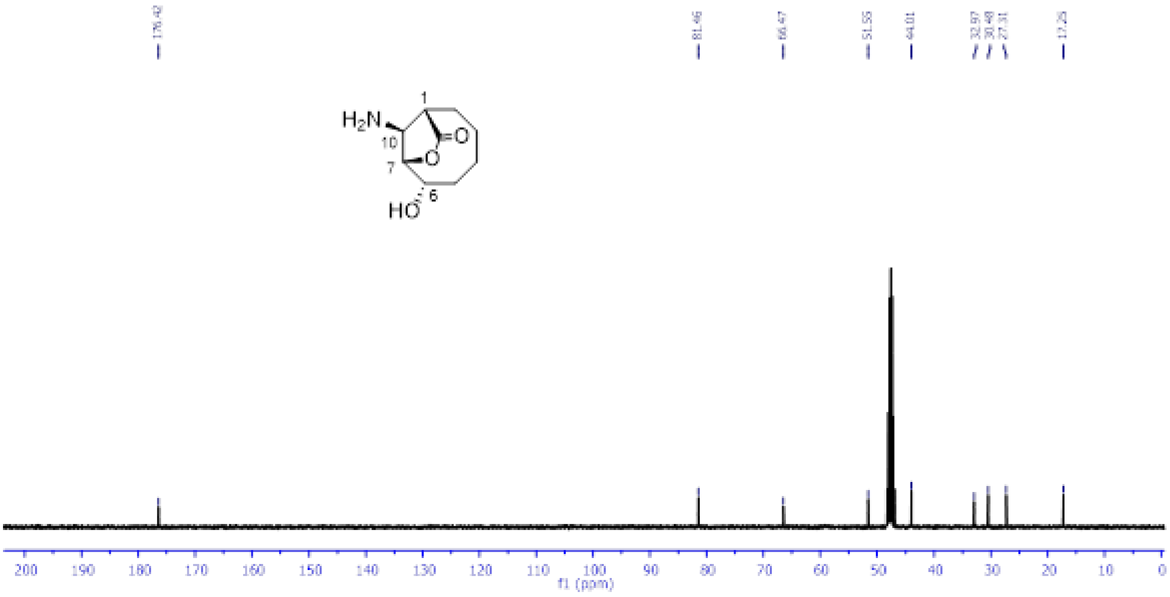

**(1*S**,6*S**,7*S**,10*S**)-10-Amino-6-hydroxy-8-oxabicyclo[5.2.1]decan-9-one (8): $CD_3OD$ (HMQC)**

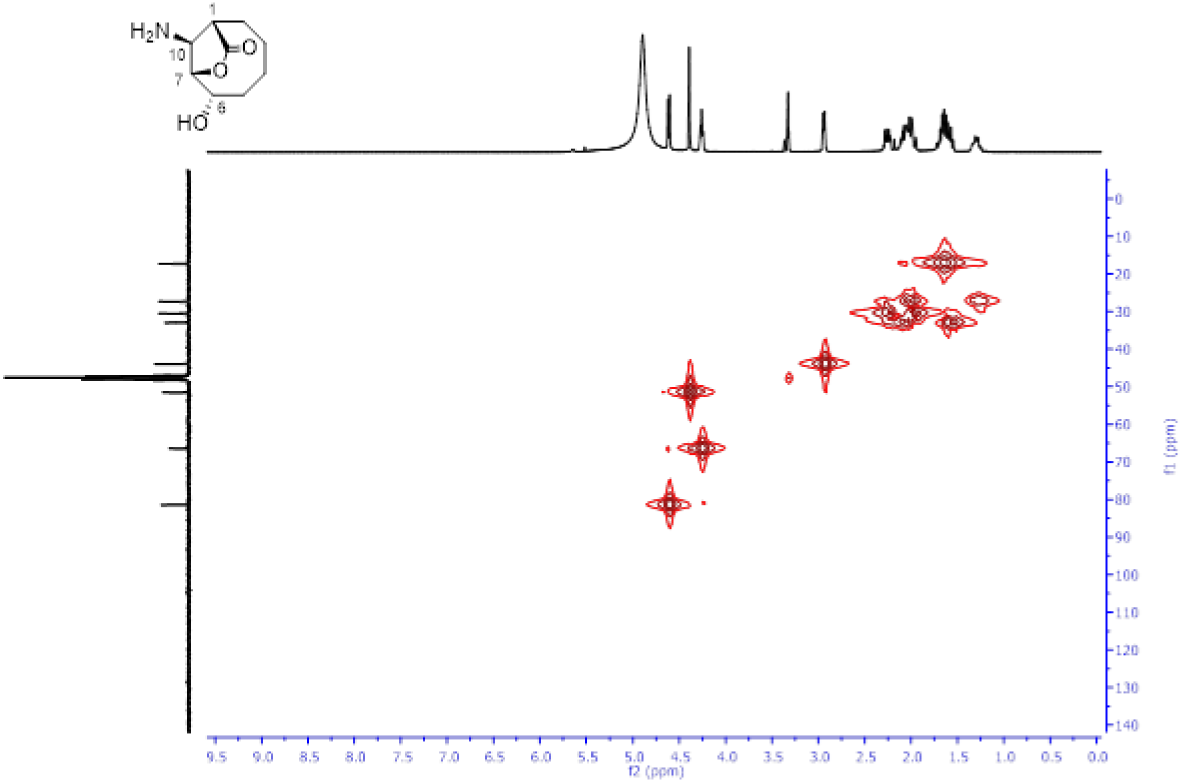


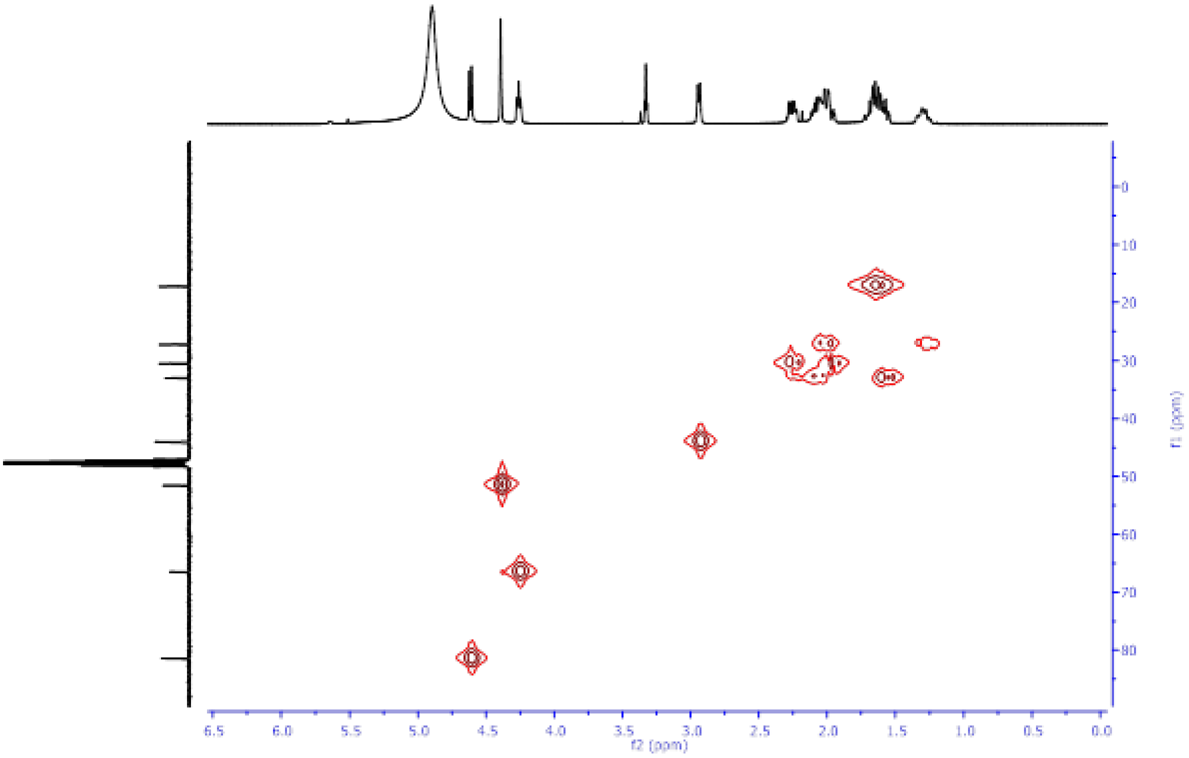

**(1*S**,6*S**,7*S**,10*S**)-10-Amino-6-hydroxy-8-oxabicyclo[5.2.1]decan-9-one (8): $CD_3OD$ (COSY)**

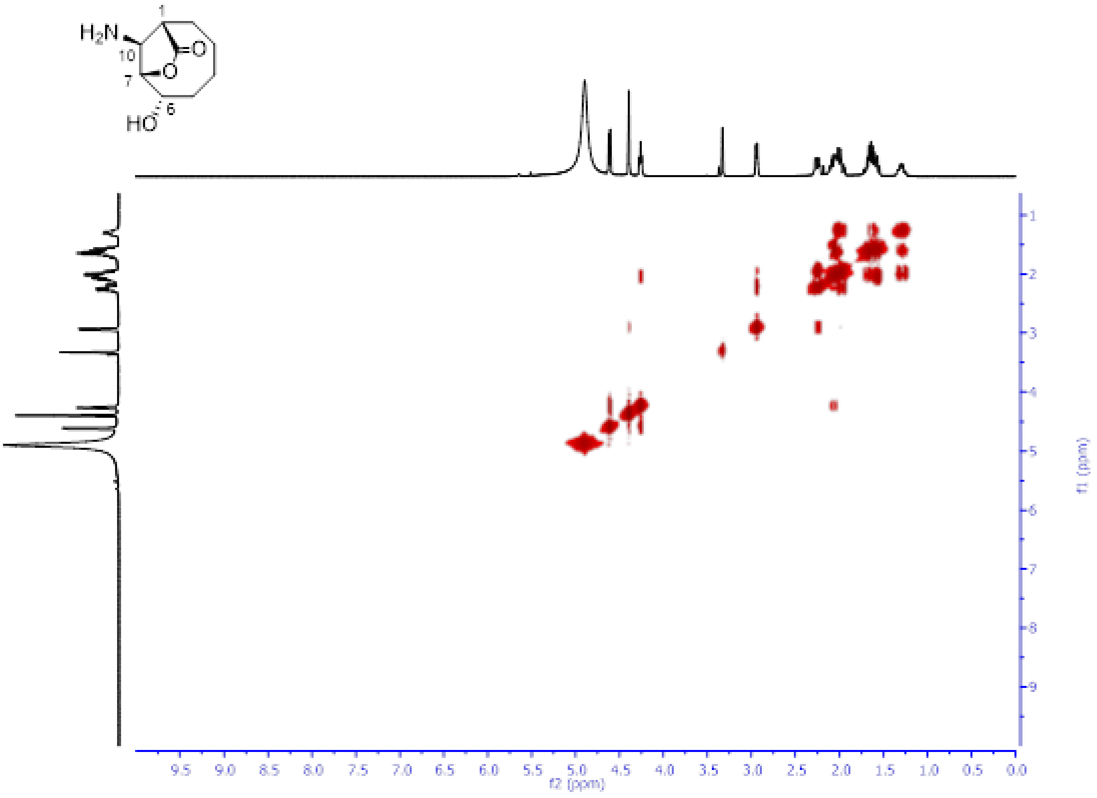


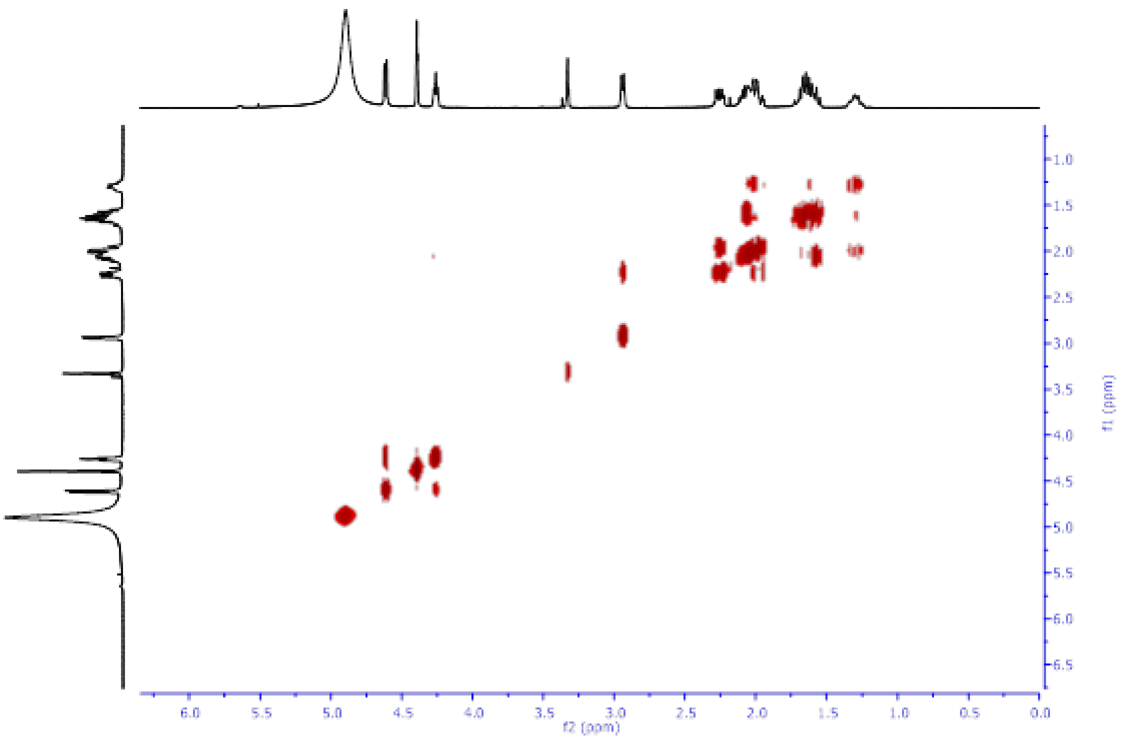

**(1*S**,6*S**,7*S**,10*S**)-10-Amino-6-hydroxy-8-oxabicyclo[5.2.1]decan-9-one (8): $CD_3OD$ (Double Resonance)**

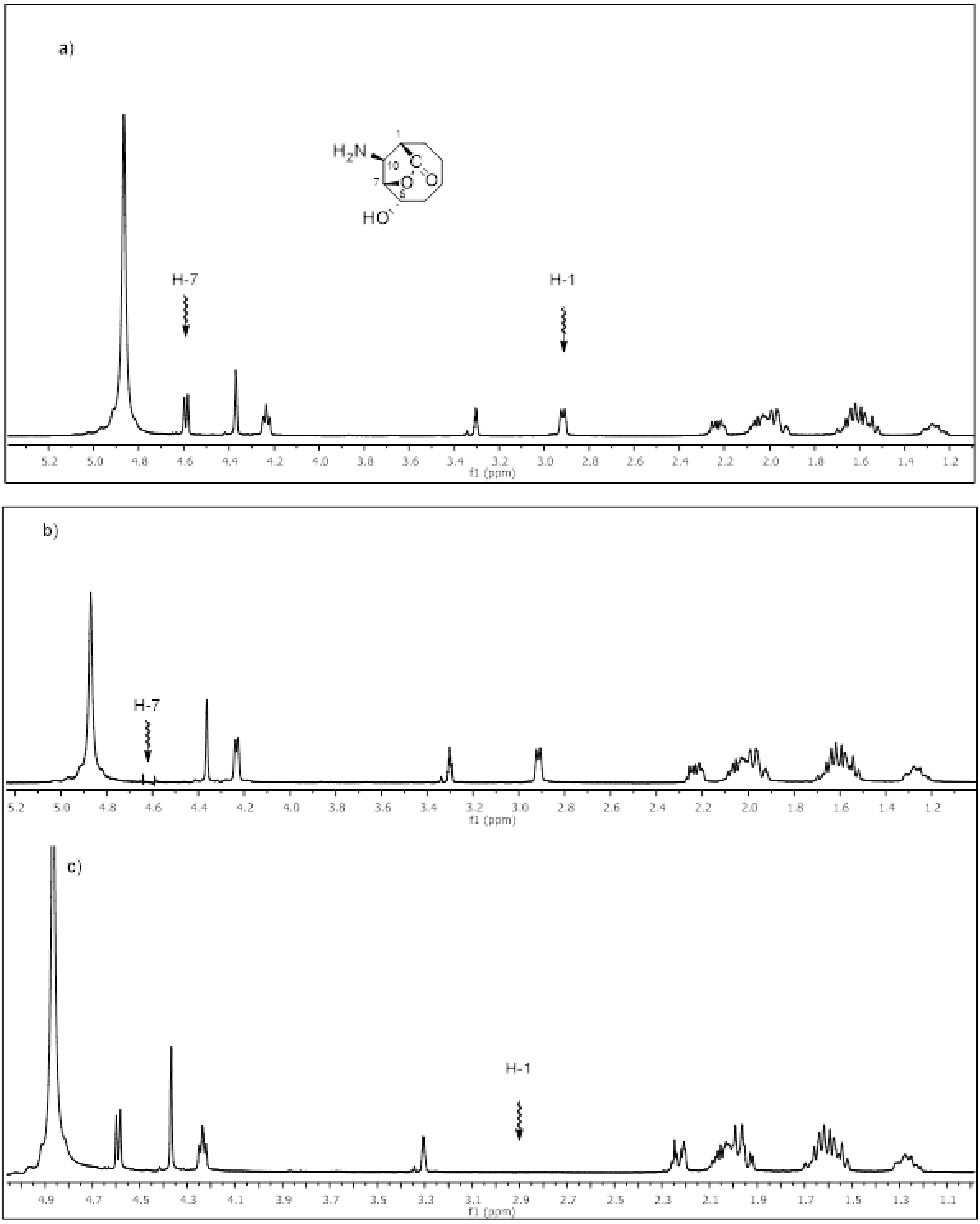

**Methyl ((1*S**,6*S**,7*S**,10*S**)-6-hydroxy-9-oxo-8-oxabicyclo[5.2.1]decan-10-yl)carbamate (10): acetone-$d_6$ ($^1$H NMR and $^{13}$C NMR)**

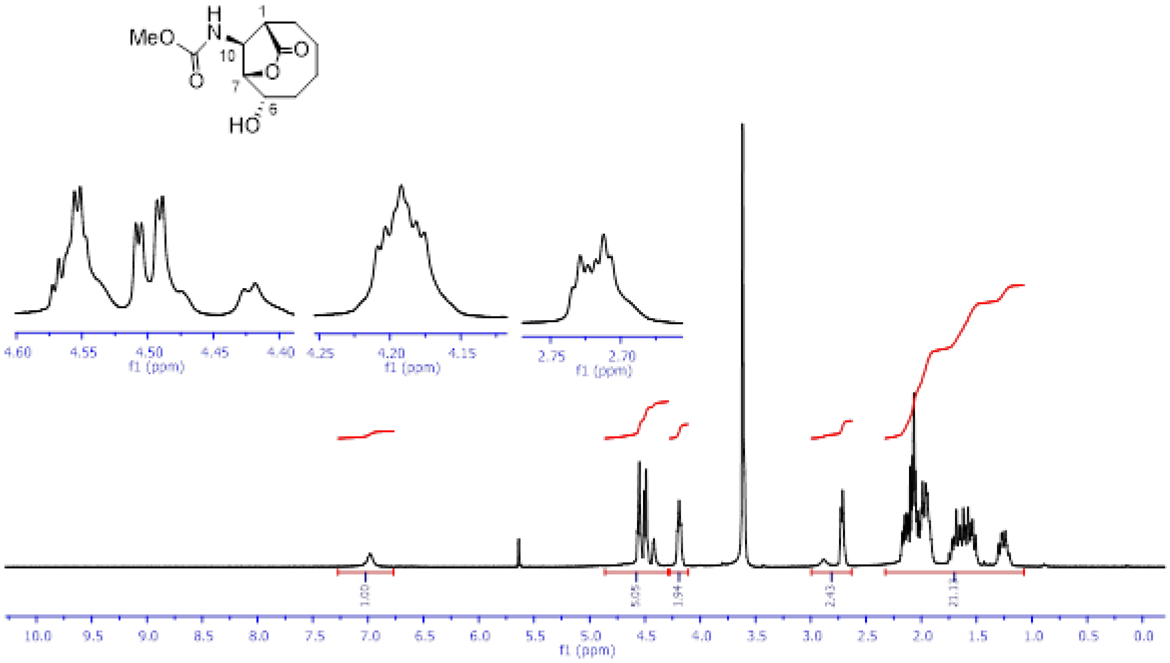

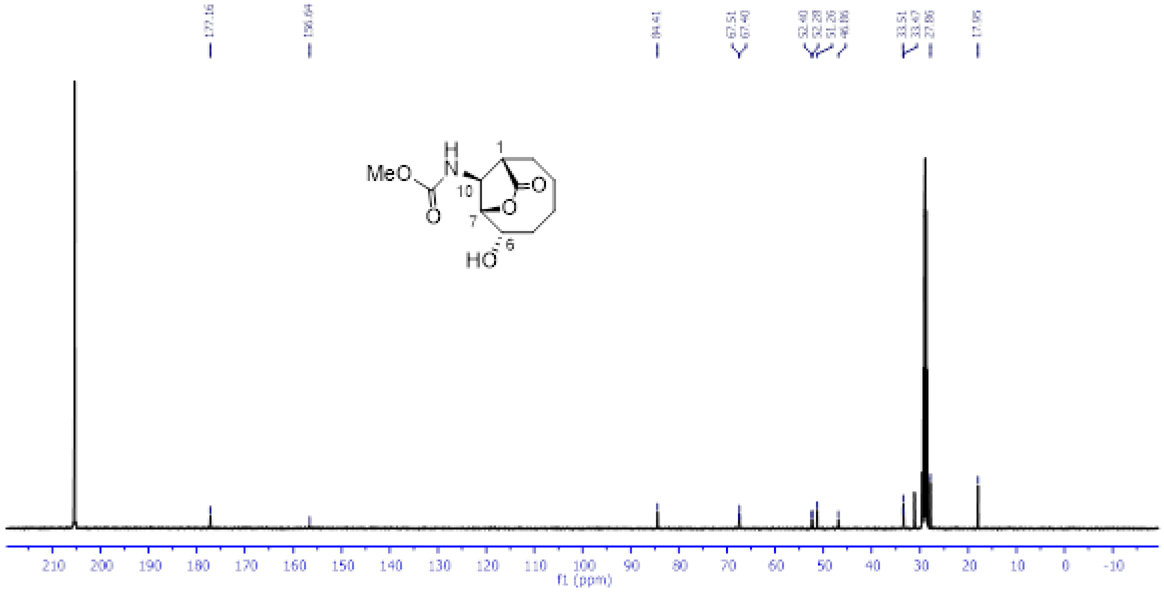

**Methyl ((1*S**,6*S**,7*S**,10*S**)-6-hydroxy-9-oxo-8-oxabicyclo[5.2.1]decan-10-yl)carbamate (10): acetone-$d_6$ (HMQC)**

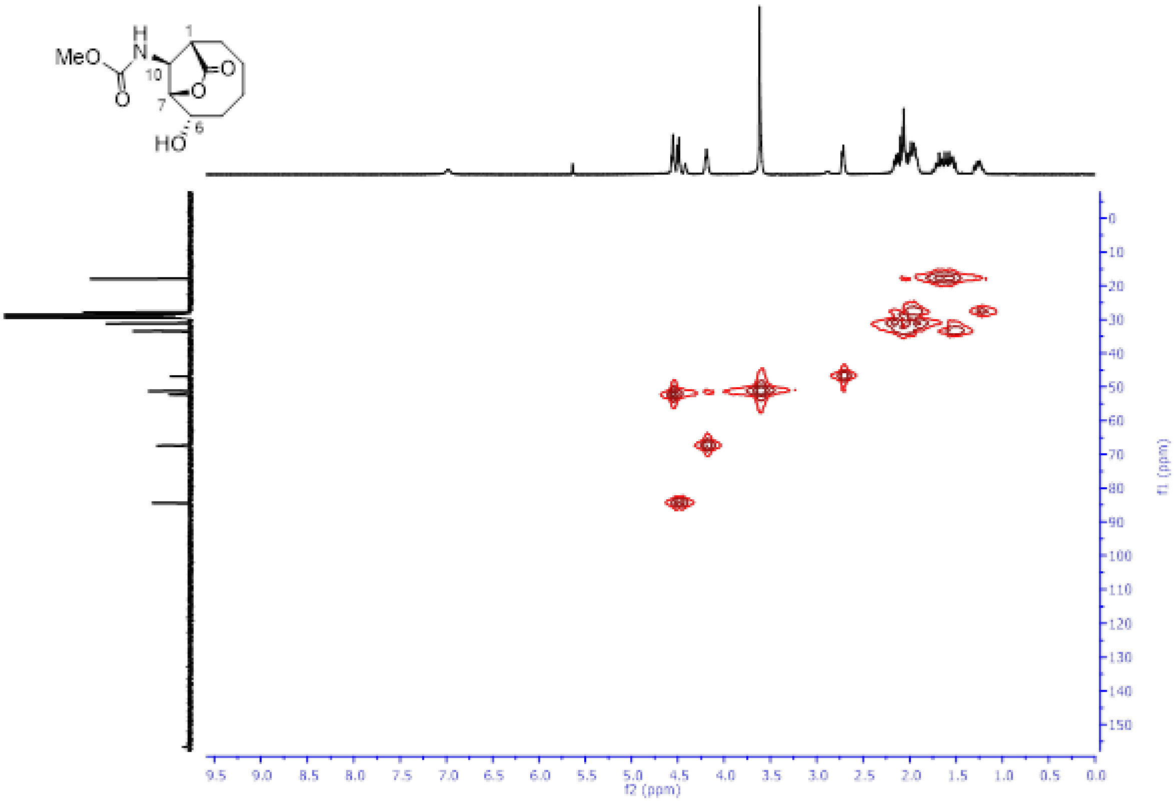


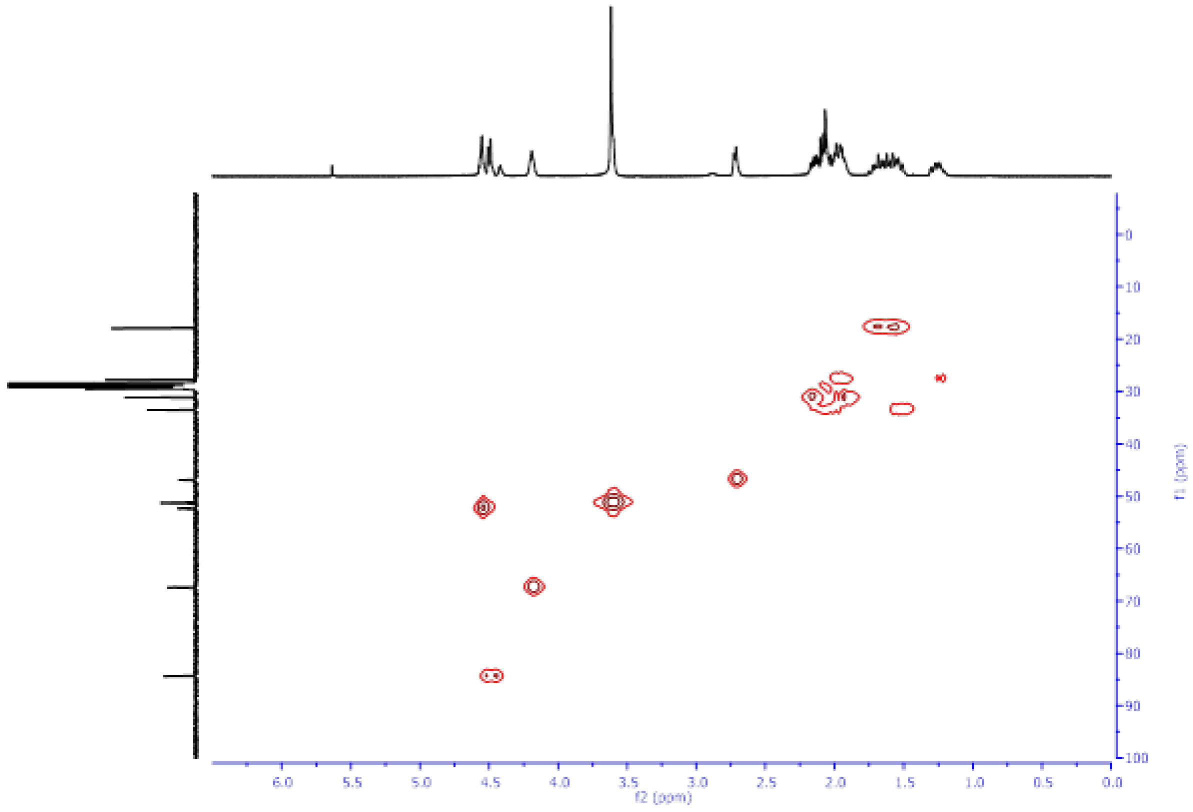

**Methyl ((1*S**,6*S**,7*S**,10*S**)-6-hydroxy-9-oxo-8-oxabicyclo[5.2.1]decan-10-yl)carbamate (10): acetone-$d_6$ (COSY)**

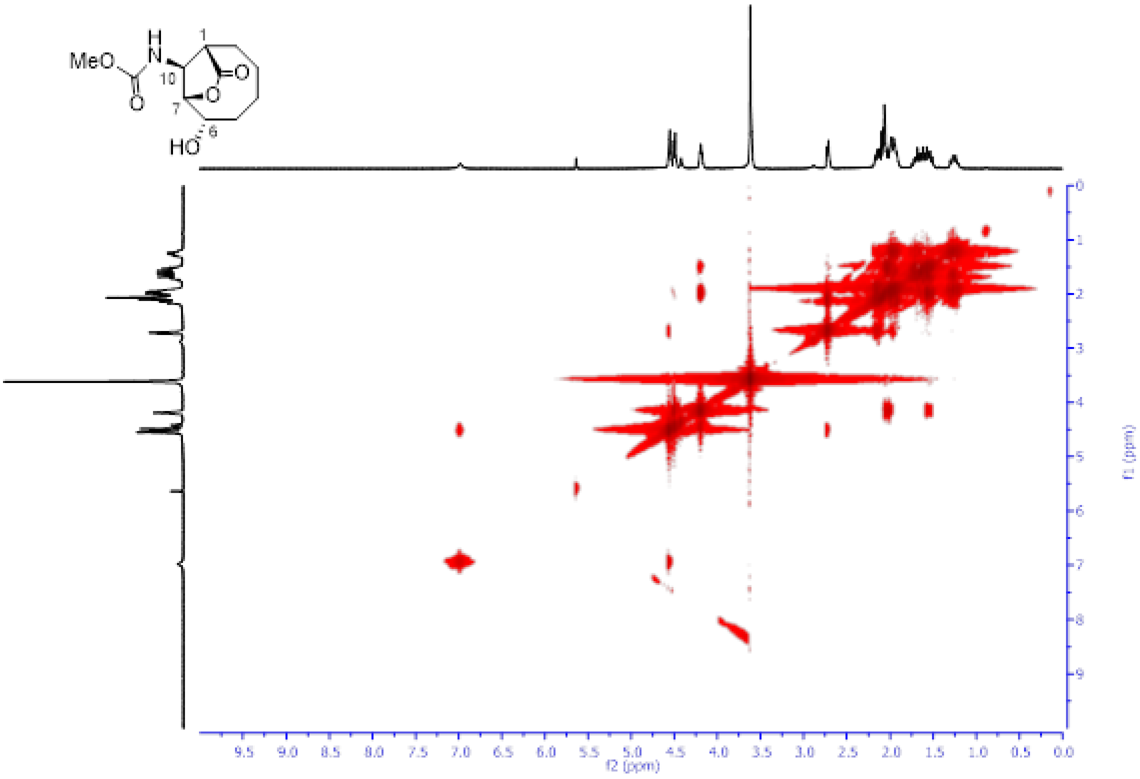


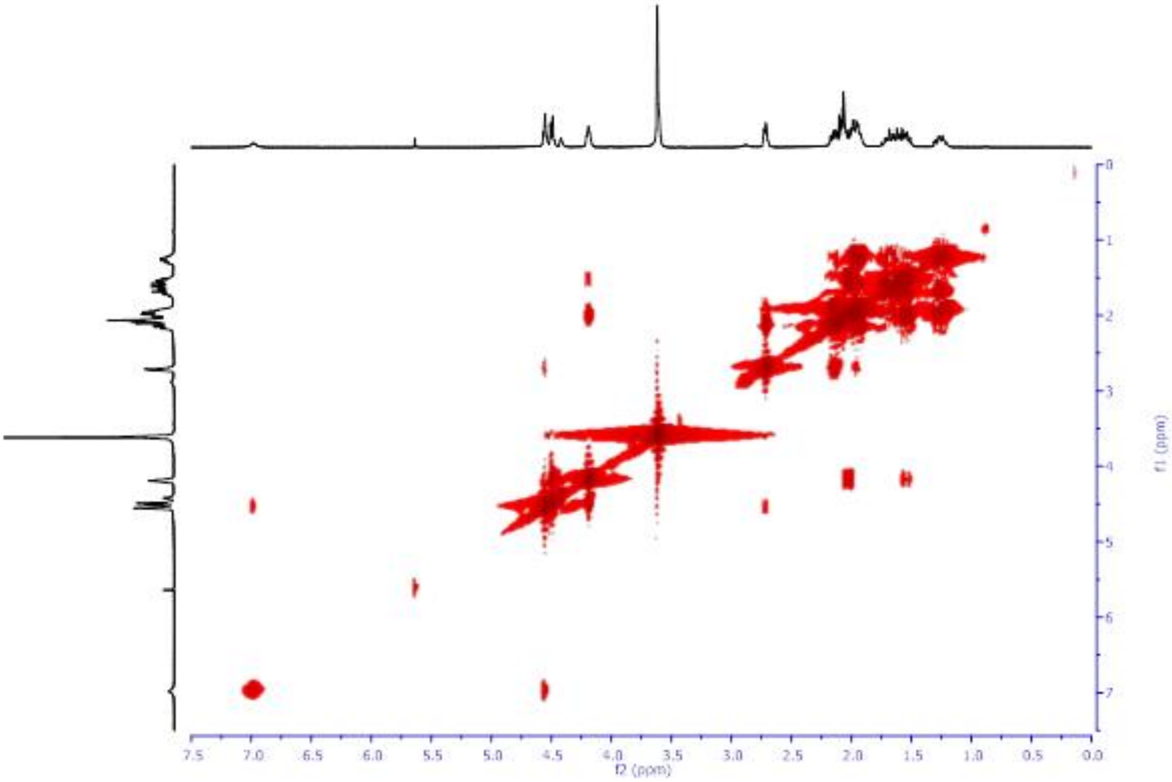

**Methyl ((1*S**,6*S**,7*S**,10*S**)-6-hydroxy-9-oxo-8-oxabicyclo[5.2.1]decan-10-yl)carbamate (10): acetone-d$_6$ (Double Resonance)**

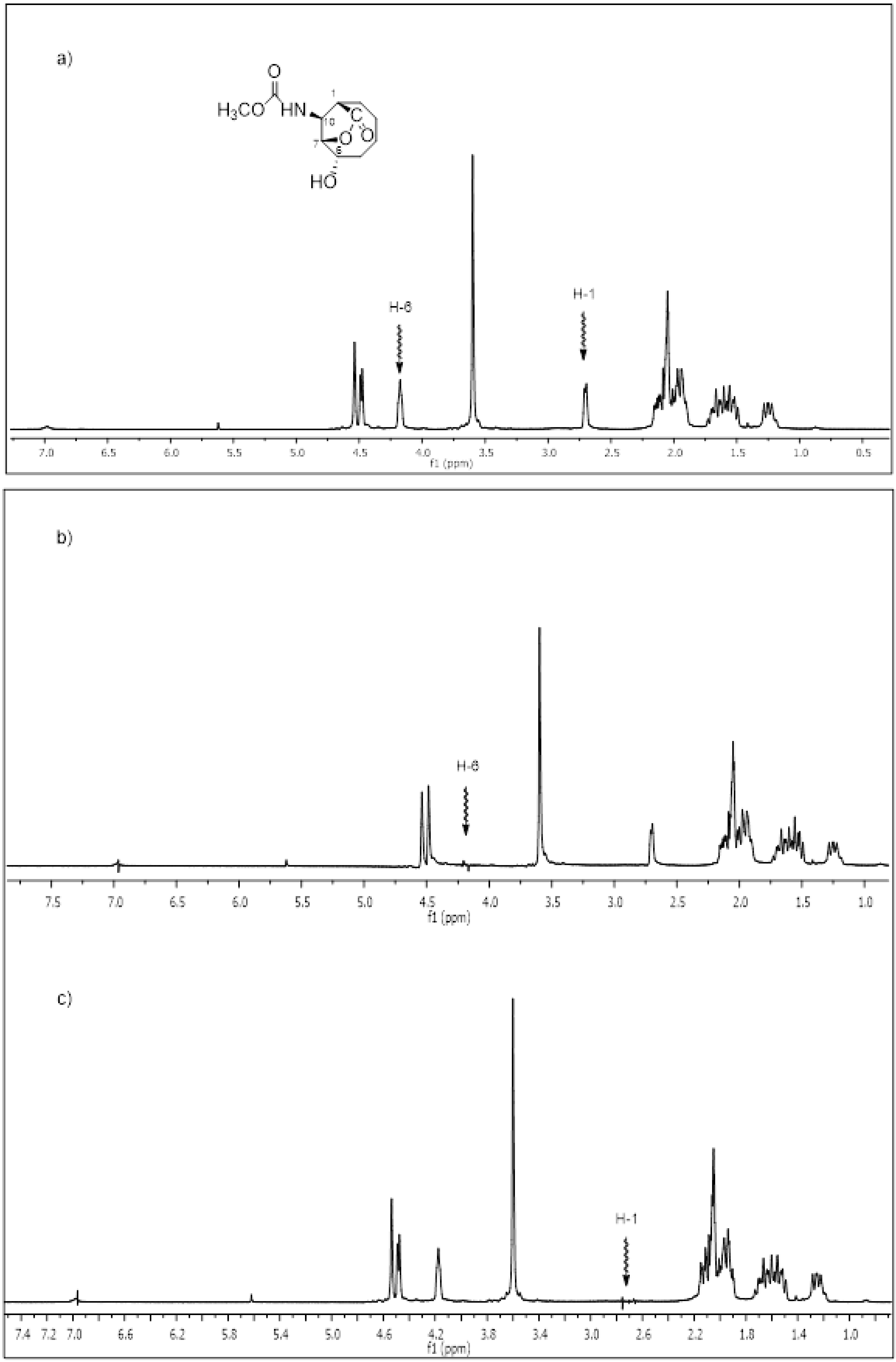

***tert*-Butyl ((1*S**,6*S**,7*S**,10*S**)-6-hydroxy-9-oxo-8-oxabicyclo[5.2.1]decan-10-yl)carbamate (13): $CDCl_3$ ($^1$H NMR and $^{13}$C NMR)**

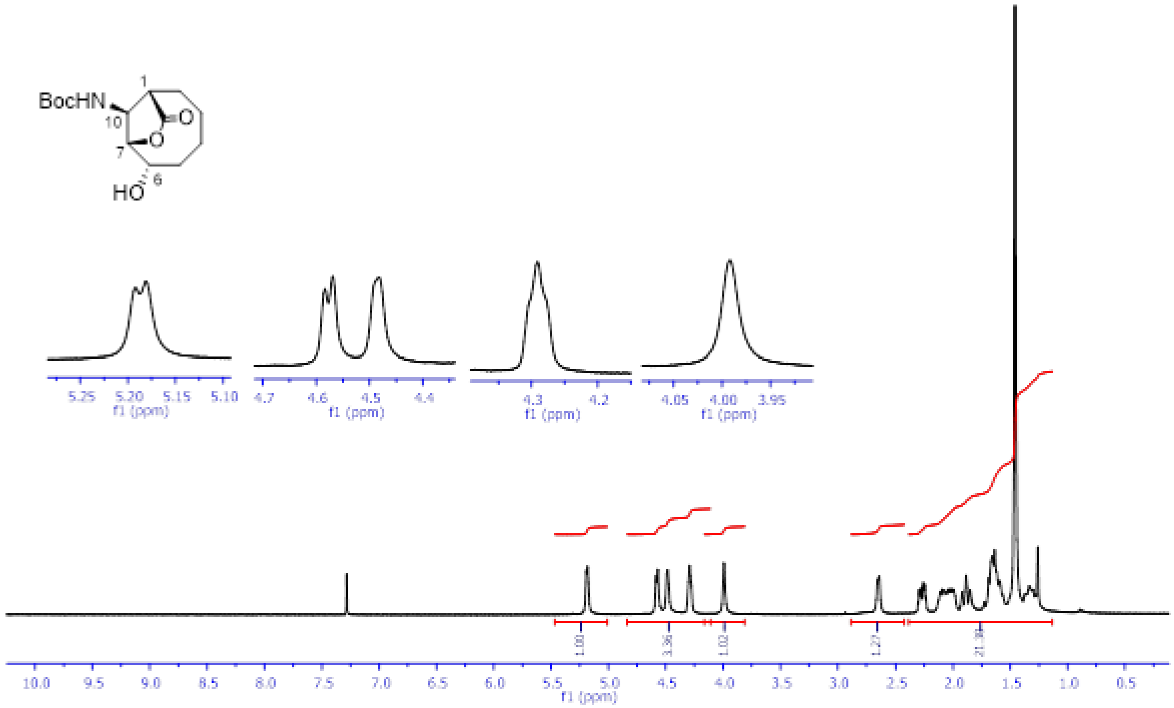


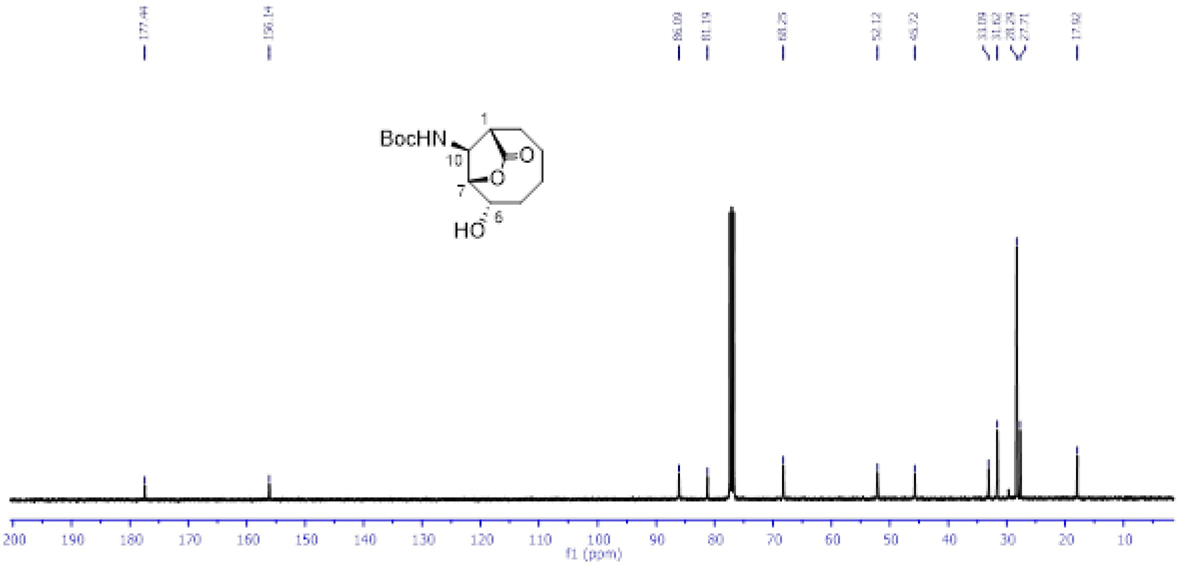

*tert*-Butyl ((1*S**,6*S**,7*S**,10*S**)-6-hydroxy-9-oxo-8-oxabicyclo[5.2.1]decan-10-yl)carbamate (13): $CDCl_3$ (HMQC)

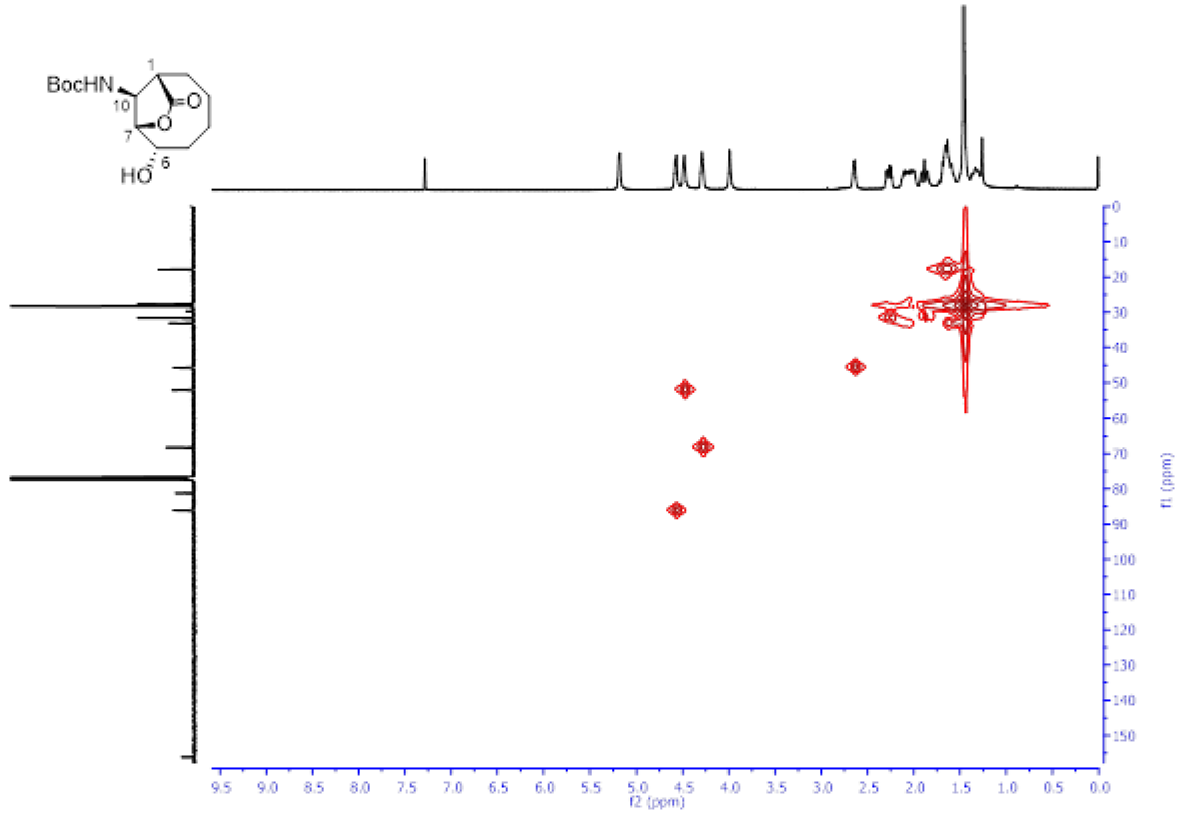


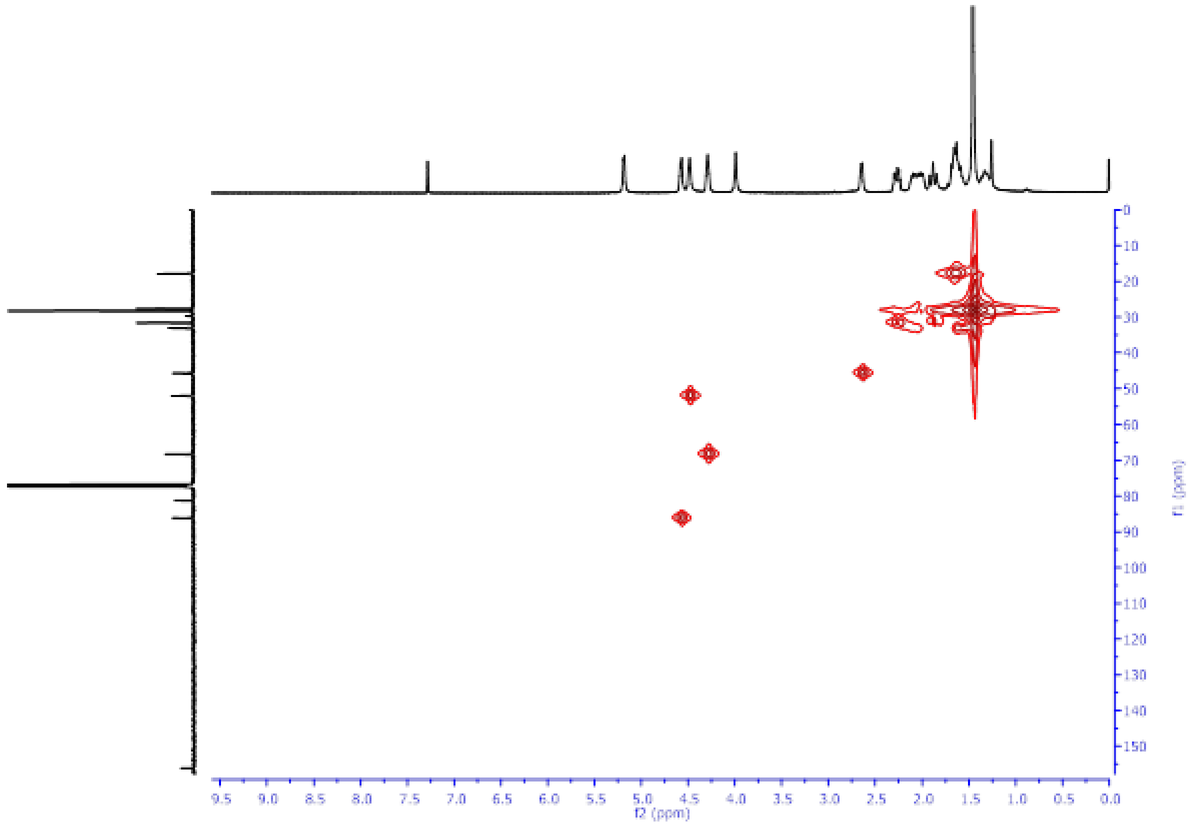

***tert*-Butyl ((1*S**,6*S**,7*S**,10*S**)-6-hydroxy-9-oxo-8-oxabicyclo[5.2.1]decan-10-yl)carbamate (13): $CDCl_3$ (COSY)**

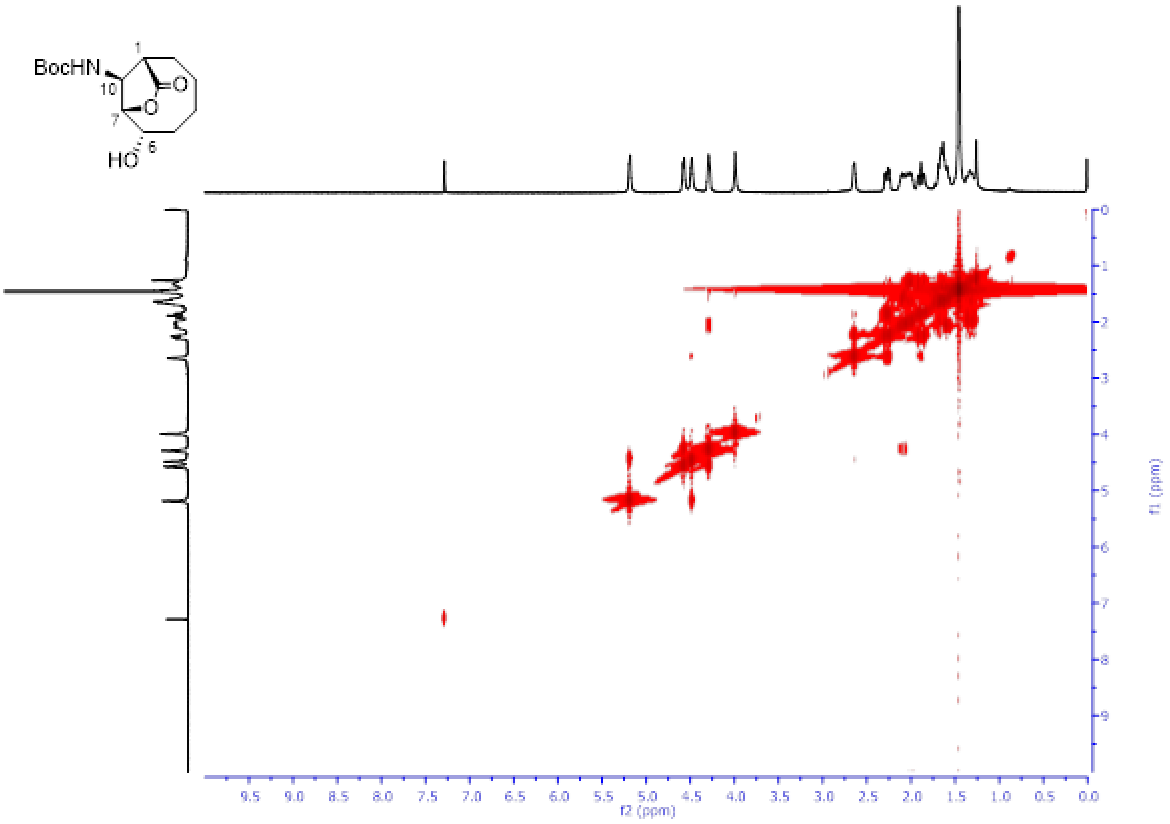


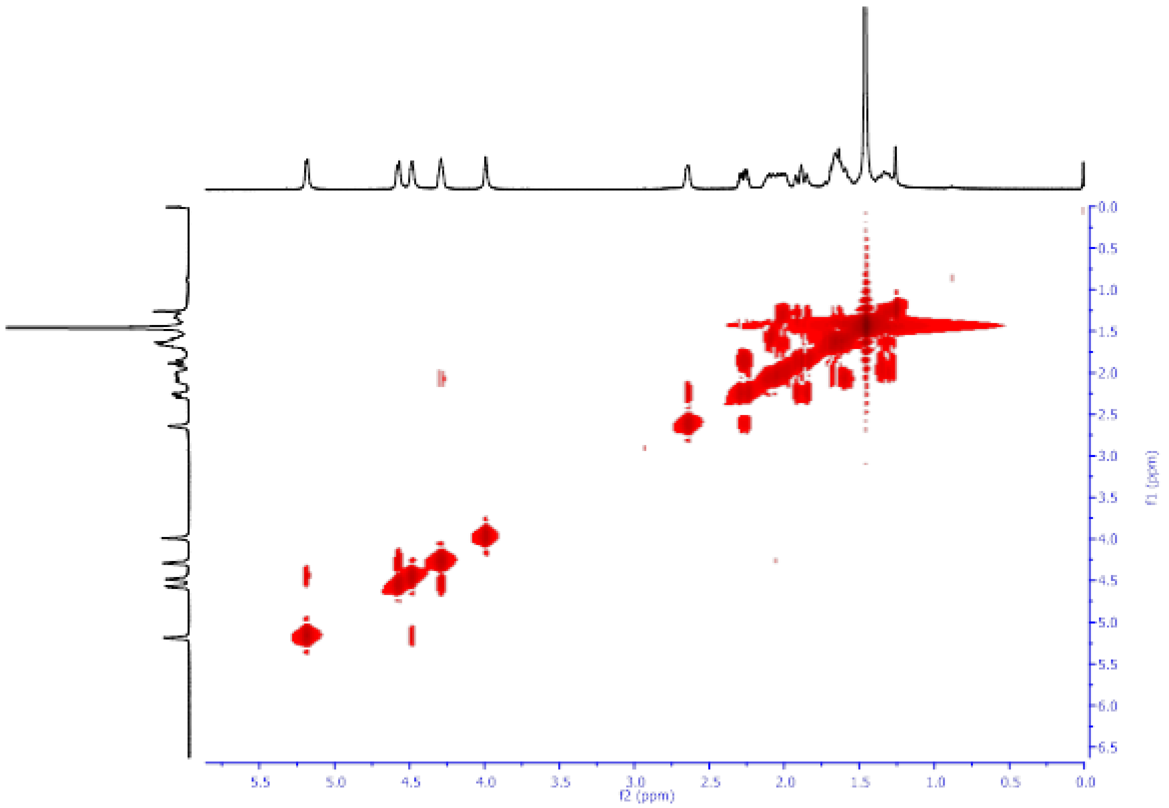

## 3. X-ray crystallographic data

For the crystal structure determination, single-crystal of the compound **10** was used for data collection on a four-circle Rigaku R-AXIS RAPID-S diffractometer (equipped with a two-dimensional area IP detector). Graphite-monochromated Mo-$K_\alpha$ radiation ($\lambda$ = 0.71073 Å) and oscillation scans technique with $\Delta w$ = 5º for one image were used for data collection. The lattice parameters were determined by the least-squares methods based on all reflections with $F^2 > 2\sigma(F^2)$. Integration of the intensities, correction for Lorentz and polarization effects and cell refinement were performed using CrystalClear (Rigaku/MSC Inc., 2005) software [3]. The structure was solved by direct methods using SHELXS-97 [4] and non-hydrogen atoms were refined using anisotropic displacement parameters by full-matrix least-squares procedure using the program SHELXL-97 [4] Hydrogen atoms were positioned geometrically and refined using a riding model. The final difference Fourier maps showed no peaks of chemical significance. Details about the analysed crystal and data collection are presented in Table S1.

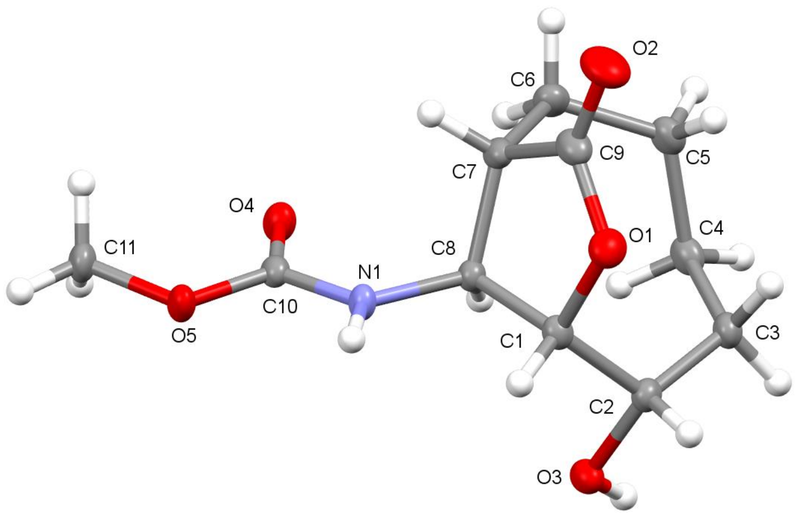


**Figure S1.** X-ray crystal structure of **10**

**Table S1.** Crystal data and structure refinement for compound **10**

| | |
|---|---|
| Empirical formula | $C_{11}H_{17}NO_5$ |
| Formula weight | 243.26 |
| Temperature | 293 K |
| Wavelength | 0.71073 A |
| Crystal system, space group | triclinic, *P*-1; (no:2) |
| Unit cell dimensions | *a* = 6.7797(6), *b* = 9.6852(7), *c* = 9.9430(7) Å, *α*= 66.480(6), *β* = 79.690(4), *γ* = 77.471(6)° |
| Volume | 581.27(8) $Å^3$ |
| Z, calculated density | 2, 1.390 $g/cm^3$ |
| absorption coefficient | 0.110 $mm^{-1}$ |
| *F*(000) | 260 |
| *θ*-range for data collection | 2.4-28.4° |
| refinement method | full matrix least-square on $F^2$ |
| data/parameters | 2672/158 |
| goodness-of-fit on $F^2$ | 1.451 |
| final *R*-indices [$I > 2\sigma(I)$] | $R_1$ = 0.101, w$R_2$ = 0.193 |
| largest diff. peak and hole | 0.267 and -0.231 e $Å^{-3}$ |

## 4. Relative free energy profile

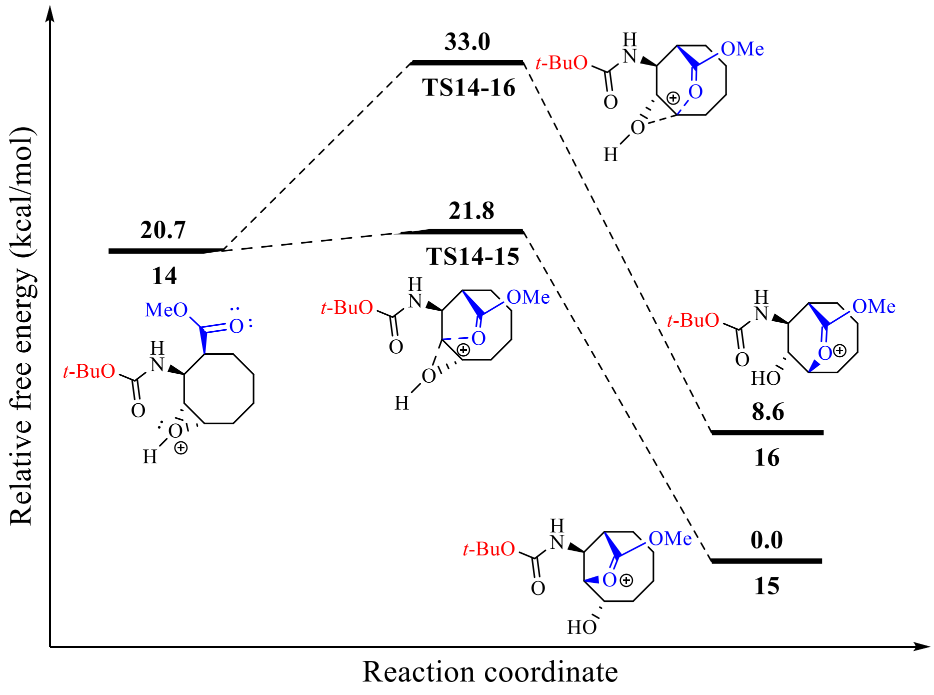


**Figure S2.** Relative free energy profile at 298.15 K for the reaction mechanism of **14** shown in Scheme 5.

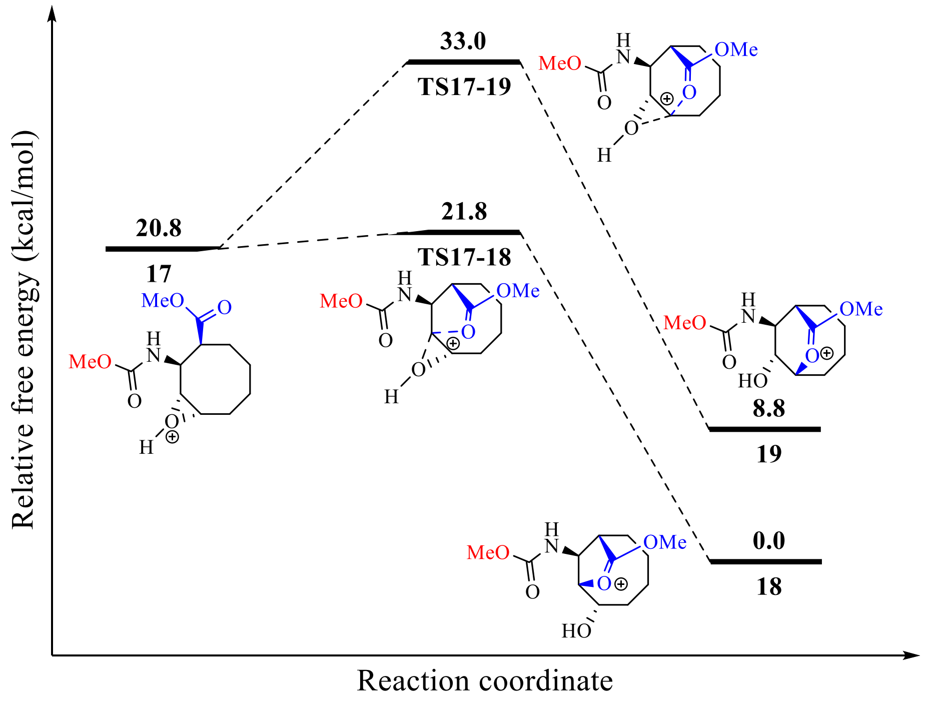


**Figure S3.** Relative free energy profile at 298.15 K for the reaction mechanism of **17** shown in Scheme 5.

## 5. The optimized geometries of the transition states with selected interatomic distances

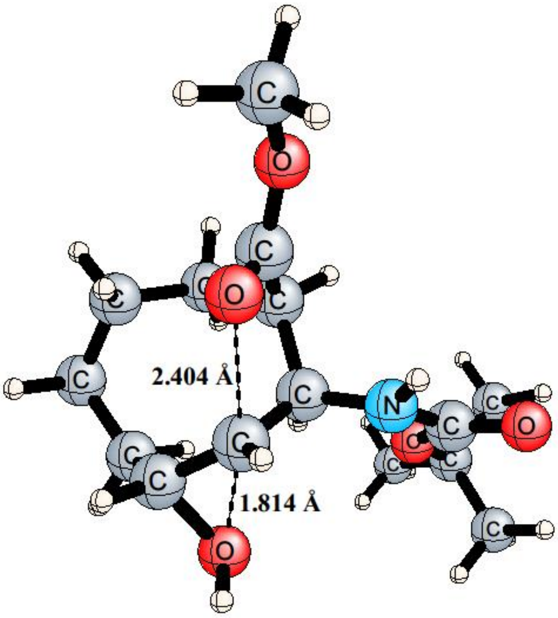


**Figure S4.** Computed structure of **TS14-15** at the B3LYP/6-311++G(d,p) level (im. freq.= 187*i*).

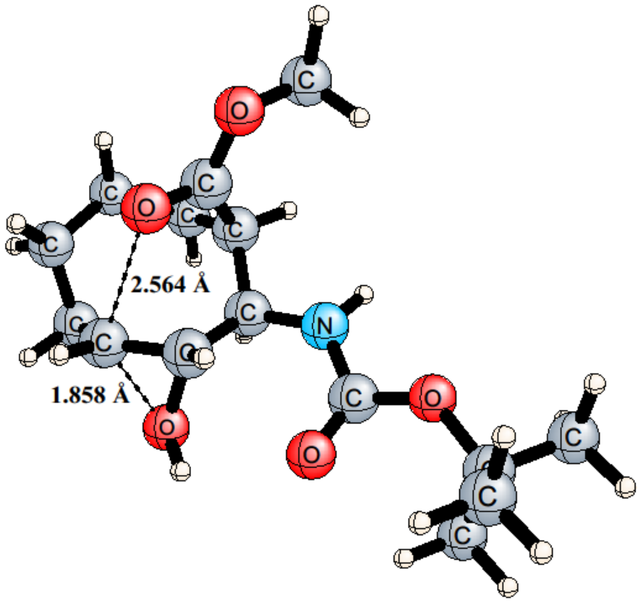


**Figure S5.** Computed structure of **TS14-16** at the B3LYP/6-311++G(d,p) level (im. freq.= 245*i*).

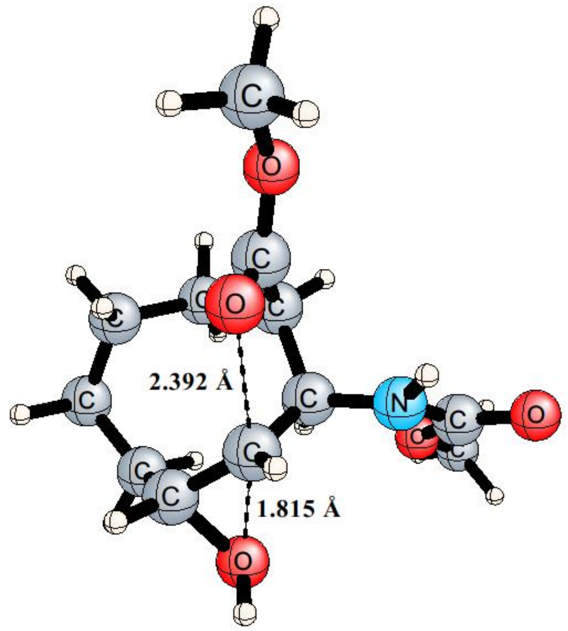


**Figure S6.** Computed structure of **TS17-18** at the B3LYP/6-311++G(d,p) level (im. freq.= 193*i*).

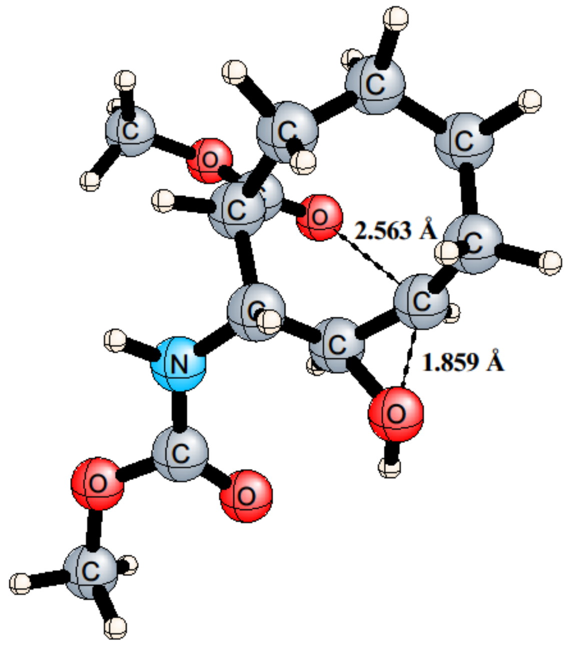


**Figure S7.** Computed structure of **TS17-19** at the B3LYP/6-311++G(d,p) level (im. freq.= 243*i*).

## 6. Cartesian coordinates for optimized structures

**7a**

E=-1017.808638 a.u., number of negative frequencies = 0

0 1

| | | | |
|---|---|---|---|
| C | 0.54100600 | 0.12653600 | -0.46070400 |
| C | 1.15057500 | 1.07151800 | -1.47881100 |
| C | 1.52142800 | 2.45235400 | -1.12892700 |
| C | 1.33550100 | 3.07018300 | 0.24081200 |
| C | 2.45021500 | 2.72979300 | 1.24431200 |
| C | 2.42450500 | 1.30832800 | 1.83709000 |
| C | 2.78929800 | 0.14750200 | 0.87829000 |
| C | 1.59542100 | -0.68686100 | 0.34228900 |
| C | 2.16030800 | -1.82968400 | -0.50009100 |
| N | -0.42945600 | -0.76640700 | -1.08815600 |
| C | -1.77972800 | -0.81242800 | -0.86015100 |
| O | -2.54309200 | -1.46780100 | -1.54056600 |
| O | -2.12311500 | -0.07485800 | 0.22276500 |
| C | -3.53705500 | 0.07953000 | 0.62534900 |
| H | -0.02048700 | 0.72015400 | 0.25517000 |
| H | 1.68453500 | 0.58891900 | -2.29274400 |
| H | 2.32172400 | 2.89965800 | -1.71976500 |
| H | 1.29243500 | 4.15697200 | 0.10935800 |
| H | 0.36217400 | 2.77795800 | 0.64436700 |
| H | 3.43011200 | 2.90784000 | 0.78311300 |
| H | 2.38107900 | 3.43788900 | 2.07676900 |
| H | 1.44448400 | 1.12359000 | 2.29403700 |
| H | 3.13968700 | 1.29477700 | 2.66537000 |
| H | 3.36877000 | 0.52561200 | 0.03143100 |
| H | 3.45759200 | -0.54105200 | 1.40448200 |
| H | 1.05919100 | -1.09945500 | 1.20032200 |
| H | -0.14338600 | -1.26255600 | -1.92062700 |
| C | -3.43619900 | 1.01724600 | 1.82941900 |
| H | -2.82328300 | 0.57115900 | 2.61643800 |
| H | -2.99066900 | 1.97134900 | 1.53865500 |
| H | -4.43169400 | 1.21081200 | 2.23597200 |
| C | -4.11531400 | -1.27546100 | 1.04099000 |
| H | -5.11927900 | -1.13139000 | 1.44996300 |
| H | -4.17615000 | -1.95451000 | 0.19222900 |
| H | -3.49614200 | -1.73184500 | 1.81820100 |
| C | -4.33743300 | 0.73451300 | -0.50308200 |
| H | -4.42130300 | 0.07380200 | -1.36429000 |
| H | -5.34238700 | 0.97142900 | -0.14277500 |
| H | -3.85976100 | 1.66614300 | -0.81672600 |

| | | | |
|---|---|---|---|
| O | 2.37926800 | -1.72303800 | -1.68138700 |
| O | 0.33491900 | 2.18071300 | -1.89172200 |
| O | 2.48463800 | -2.99892700 | 0.10398300 |
| C | 2.14191700 | -3.29251300 | 1.46581100 |
| H | 1.05873400 | -3.31138800 | 1.60430100 |
| H | 2.54326000 | -4.28702200 | 1.65175400 |
| H | 2.59934200 | -2.58605600 | 2.16195500 |

**7b**

E=-1017.7920927 a.u., number of negative frequencies = 0

0 1

| | | | |
|---|---|---|---|
| C | -0.50461000 | -0.20316300 | 0.02135100 |
| C | -0.93677500 | 0.45715200 | 1.31800200 |
| C | -1.06904400 | 1.92268600 | 1.42178500 |
| C | -0.70827900 | 2.88923100 | 0.30854000 |
| C | -1.76970900 | 3.07249900 | -0.79072000 |
| C | -1.97031400 | 1.89352300 | -1.76406500 |
| C | -2.66371000 | 0.64621100 | -1.15991200 |
| C | -1.73554400 | -0.55663400 | -0.88067000 |
| C | -2.40236800 | -1.87981200 | -0.44158000 |
| N | 0.36119400 | -1.35241400 | 0.23649800 |
| C | 1.72983600 | -1.37104200 | 0.14507600 |
| O | 2.38663300 | -2.37716600 | 0.32017800 |
| O | 2.22188900 | -0.15090200 | -0.16791400 |
| C | 3.67756800 | 0.09926000 | -0.20863900 |
| H | 0.08517900 | 0.51941300 | -0.53603600 |
| H | -1.54801500 | -0.16571100 | 1.97226800 |
| H | -1.79860400 | 2.30082300 | 2.13911900 |
| H | -0.51433600 | 3.86174700 | 0.77454900 |
| H | 0.24054900 | 2.57735000 | -0.13521800 |
| H | -2.73630200 | 3.32429500 | -0.33504300 |
| H | -1.48373000 | 3.95039500 | -1.37938800 |
| H | -1.01089900 | 1.62095400 | -2.21892600 |
| H | -2.58949800 | 2.25886600 | -2.58864300 |
| H | -3.18761100 | 0.96230500 | -0.26026500 |
| H | -3.43522400 | 0.29881900 | -1.85430900 |
| H | -1.29440000 | -0.85095500 | -1.83659400 |
| H | -0.04611300 | -2.27498600 | 0.30483400 |
| C | 3.74641000 | 1.59165500 | -0.53617000 |
| H | 3.26754800 | 1.79849700 | -1.49654200 |
| H | 3.24642900 | 2.17759300 | 0.23813400 |
| H | 4.78916000 | 1.91261500 | -0.59615000 |
| C | 4.32432200 | -0.73051500 | -1.32057000 |
| H | 5.37493700 | -0.44405400 | -1.42228800 |
| H | 4.26978300 | -1.79522100 | -1.10030600 |

| | | | |
|---|---|---|---|
| H | 3.82763200 | -0.54032100 | -2.27590300 |
| C | 4.29469600 | -0.17992900 | 1.16402300 |
| H | 4.25285000 | -1.24027800 | 1.40797100 |
| H | 5.34095700 | 0.13833500 | 1.16150700 |
| H | 3.76785600 | 0.38411900 | 1.93769600 |
| O | -1.90922700 | -2.92339100 | -0.79533500 |
| O | 0.02909100 | 1.22831800 | 2.03784200 |
| O | -3.49636700 | -1.97676300 | 0.35647200 |
| C | -4.29086400 | -0.92603200 | 0.92162000 |
| H | -4.88968600 | -0.42956500 | 0.15724600 |
| H | -4.95378300 | -1.43107100 | 1.62254100 |
| H | -3.68886700 | -0.19837000 | 1.46589500 |

**10**

E= -860.5564218 a.u., number of negative frequencies = 0

0 1

| | | | |
|---|---|---|---|
| C | -0.26507400 | 0.14434800 | -0.14799500 |
| C | 0.70908000 | 0.68185600 | -1.21832700 |
| C | 1.75128800 | 1.70077700 | -0.73211600 |
| C | 2.86274100 | 1.20346300 | 0.20354900 |
| C | 2.49966700 | 0.64728300 | 1.59892000 |
| C | 2.33310600 | -0.88510100 | 1.70859700 |
| C | 0.93649800 | -1.49216200 | 1.50048800 |
| C | 0.24825900 | -1.28358600 | 0.13471900 |
| N | -1.63123200 | 0.17672000 | -0.65508900 |
| O | 0.97238900 | 2.75433300 | -0.15585200 |
| C | -2.69820700 | 0.10986100 | 0.18692500 |
| O | -2.63112400 | 0.03965100 | 1.39703300 |
| C | 1.17215800 | -1.59448800 | -1.03536900 |
| O | 1.37957300 | -0.46425900 | -1.77947000 |
| O | 1.68350500 | -2.63864000 | -1.32245500 |
| O | -3.85817200 | 0.13617900 | -0.51851700 |
| C | -5.05950900 | 0.07958100 | 0.26802400 |
| H | -0.24405800 | 0.76520400 | 0.74429700 |
| H | 0.15482600 | 1.15752200 | -2.03004500 |
| H | 2.25158200 | 2.06860000 | -1.63819700 |
| H | 3.52395200 | 2.06822000 | 0.34150700 |
| H | 3.46326300 | 0.47032900 | -0.34552900 |
| H | 3.32389200 | 0.92154000 | 2.26386000 |
| H | 1.62252000 | 1.16603300 | 1.99959800 |
| H | 2.64094000 | -1.18279700 | 2.71627000 |
| H | 3.04602700 | -1.37151100 | 1.03228800 |
| H | 1.02116500 | -2.57099800 | 1.66010900 |
| H | 0.24784000 | -1.11602300 | 2.26392800 |
| H | -0.58843400 | -1.98301100 | 0.08196900 |

| | | | |
|---|---|---|---|
| H | -1.81401500 | 0.17843200 | -1.64683800 |
| H | 1.54769300 | 3.50303800 | 0.02969700 |
| H | -5.87525400 | 0.10134500 | -0.45159400 |
| H | -5.09260000 | -0.83947300 | 0.85477500 |
| H | -5.12012700 | 0.93741000 | 0.93912900 |

**14**

E= -1018.158489 a.u., number of negative frequencies = 0

1 1

| | | | |
|---|---|---|---|
| C | 0.78089300 | -0.24886300 | -0.55759600 |
| C | 1.02946400 | -0.73343200 | 0.85197700 |
| C | 2.11582200 | -1.58209500 | 1.37529000 |
| C | 3.28283600 | -2.20380100 | 0.66534600 |
| C | 3.31392100 | -2.16424600 | -0.86893500 |
| C | 3.77098800 | -0.82102200 | -1.48303200 |
| C | 3.27373700 | 0.48086100 | -0.83148700 |
| C | 1.78290100 | 0.84653700 | -1.00795100 |
| N | -0.57955000 | 0.27932900 | -0.65375300 |
| O | 0.75110300 | -2.23198100 | 0.99759700 |
| C | -1.69587500 | -0.32906300 | -0.11145200 |
| O | -2.78999500 | 0.18677200 | -0.64833300 |
| O | -1.63939300 | -1.18905100 | 0.76057400 |
| C | -4.17976500 | -0.17852400 | -0.19693300 |
| C | -4.42426100 | -1.65962800 | -0.47584400 |
| C | -5.04407300 | 0.70596600 | -1.09053100 |
| C | -4.34566900 | 0.18825500 | 1.27590900 |
| H | 0.04887000 | -2.34605100 | 1.66988300 |
| C | 1.49365000 | 2.18356300 | -0.32428300 |
| O | 1.22579200 | 3.19964300 | -0.90645600 |
| O | 1.60233400 | 2.08227600 | 1.01857400 |
| C | 1.38604400 | 3.30840100 | 1.76776200 |
| H | 0.90484900 | -1.10302100 | -1.22775200 |
| H | 0.49291500 | -0.17910300 | 1.61009500 |
| H | 2.21204900 | -1.54948200 | 2.45713800 |
| H | 3.34146700 | -3.23689100 | 1.02558300 |
| H | 4.16979200 | -1.70983900 | 1.08364600 |
| H | 4.01675300 | -2.93385900 | -1.19709400 |
| H | 2.34721000 | -2.48646700 | -1.26273300 |
| H | 4.86389400 | -0.78845000 | -1.43335700 |
| H | 3.52581900 | -0.82097700 | -2.54991000 |
| H | 3.84637900 | 1.30432600 | -1.26742500 |
| H | 3.52554200 | 0.49345700 | 0.23326600 |
| H | 1.59082400 | 1.01898400 | -2.07029500 |
| H | -0.76567000 | 0.92133500 | -1.41269100 |
| H | -5.47167000 | -1.89161900 | -0.26834000 |

| | | | |
|---|---|---|---|
| H | -3.80141900 | -2.29655400 | 0.15078900 |
| H | -4.23277000 | -1.89046800 | -1.52641200 |
| H | -6.09858300 | 0.53357900 | -0.86524200 |
| H | -4.82470000 | 1.76228200 | -0.92329100 |
| H | -4.87615400 | 0.47650400 | -2.14456800 |
| H | -4.09494300 | 1.23864800 | 1.44262200 |
| H | -5.39084500 | 0.04574100 | 1.56062400 |
| H | -3.72866300 | -0.43676400 | 1.92029200 |
| H | 1.52621400 | 3.03972700 | 2.81165200 |
| H | 2.11072000 | 4.06198900 | 1.46212400 |
| H | 0.37657000 | 3.67893700 | 1.59257800 |

**15**

E= -1018.1936757 a.u., number of negative frequencies = 0

1 1

| | | | |
|---|---|---|---|
| C | -0.40957400 | -0.46480500 | -0.05794800 |
| C | -1.48079100 | -0.60402200 | -1.14957400 |
| C | -2.60360000 | -1.65135100 | -0.97898400 |
| C | -2.87673100 | -2.18631000 | 0.43567100 |
| C | -3.71574800 | -1.33992000 | 1.40812000 |
| C | -3.32363900 | 0.12196300 | 1.69206100 |
| C | -1.84692500 | 0.44889900 | 1.99958200 |
| C | -0.90643400 | 0.74163200 | 0.78469100 |
| N | 0.87377500 | -0.15342800 | -0.67632400 |
| O | -2.11890100 | -2.69832500 | -1.82144800 |
| C | 2.00047800 | -0.10263400 | 0.14492700 |
| O | 3.09968600 | -0.14865700 | -0.59774100 |
| O | 1.91103900 | -0.00823600 | 1.35303900 |
| C | -1.66260800 | 1.49402600 | -0.25014800 |
| O | -2.05533500 | 0.78885100 | -1.24796100 |
| C | 4.47623000 | -0.05972700 | 0.00018100 |
| C | 4.63288600 | 1.28407300 | 0.70907300 |
| C | 5.36711400 | -0.14318300 | -1.23578500 |
| C | 4.69993700 | -1.25118100 | 0.92928100 |
| O | -1.95536000 | 2.72872000 | -0.16185100 |
| C | -2.74407500 | 3.38983900 | -1.21949200 |
| H | -0.34260700 | -1.36099000 | 0.55863700 |
| H | -1.06145000 | -0.76476100 | -2.14023400 |
| H | -3.52097600 | -1.21803200 | -1.40161700 |
| H | -3.42695800 | -3.12410400 | 0.29935200 |
| H | -1.93077300 | -2.48317200 | 0.89558300 |
| H | -4.75599300 | -1.33515700 | 1.06631200 |
| H | -3.72245100 | -1.88288000 | 2.35831100 |
| H | -3.90883500 | 0.43628600 | 2.56020700 |
| H | -3.68909800 | 0.76210500 | 0.87903800 |

| | | | |
|---|---|---|---|
| H | -1.81827300 | 1.34609300 | 2.62116600 |
| H | -1.37206300 | -0.34252900 | 2.58541200 |
| H | -0.05477200 | 1.31283200 | 1.14893600 |
| H | 1.04714300 | -0.51936800 | -1.60301900 |
| H | -2.67139800 | -3.48165400 | -1.72350500 |
| H | 5.67133200 | 1.39760700 | 1.02955200 |
| H | 3.99415400 | 1.35205700 | 1.58878500 |
| H | 4.40119200 | 2.10768500 | 0.02910500 |
| H | 6.41584600 | -0.09103700 | -0.93580800 |
| H | 5.20849200 | -1.08375000 | -1.76719000 |
| H | 5.16347000 | 0.68363000 | -1.91932500 |
| H | 4.51892600 | -2.19062500 | 0.40128000 |
| H | 5.74028800 | -1.25074300 | 1.26356000 |
| H | 4.05755800 | -1.20323300 | 1.80755900 |
| H | -2.78864400 | 4.42798300 | -0.90840800 |
| H | -2.22443400 | 3.27718400 | -2.16865300 |
| H | -3.73425800 | 2.93993300 | -1.25302000 |

**16**

E= -1018.1822621 a.u., number of negative frequencies = 0

1 1

| | | | |
|---|---|---|---|
| C | -0.39773700 | -0.26945100 | 0.45502100 |
| C | -0.67591800 | -1.24517300 | -0.73420400 |
| C | -2.17295600 | -1.34545700 | -1.02816300 |
| C | -3.01416700 | -1.99242800 | 0.08925000 |
| C | -4.27231700 | -1.24686500 | 0.55753600 |
| C | -4.05073300 | 0.11745500 | 1.27006600 |
| C | -2.62295800 | 0.44515100 | 1.74554300 |
| C | -1.53501500 | 0.79158900 | 0.63760200 |
| N | 0.85854800 | 0.46010000 | 0.25734000 |
| C | 2.06923600 | -0.20134200 | 0.09335000 |
| O | 3.06917200 | 0.64703300 | 0.28887900 |
| O | 2.15587600 | -1.38049000 | -0.20676700 |
| C | -2.24570800 | 1.02334200 | -0.65382600 |
| O | -2.62332800 | 0.04615500 | -1.37783000 |
| O | -0.25985400 | -2.55647500 | -0.48430400 |
| C | 4.51354000 | 0.25317700 | 0.10848100 |
| C | 4.86803100 | -0.83592100 | 1.11821200 |
| C | 5.23787300 | 1.55900500 | 0.42129000 |
| C | 4.74102500 | -0.17690300 | -1.33877100 |
| O | -2.60231700 | 2.16577500 | -1.12351900 |
| C | -2.29954700 | 3.43449000 | -0.46201900 |
| H | -0.35786800 | -0.87285400 | 1.36736200 |
| H | -0.18509400 | -0.82755600 | -1.62356700 |
| H | -2.32615800 | -1.84954800 | -1.97810200 |

| | | | |
|---|---|---|---|
| H | -3.31581100 | -2.97414300 | -0.27972200 |
| H | -2.36331400 | -2.19983000 | 0.94014300 |
| H | -4.78020900 | -1.92203200 | 1.24957200 |
| H | -4.96060900 | -1.10970600 | -0.28004900 |
| H | -4.68622700 | 0.15288100 | 2.15791200 |
| H | -4.41956900 | 0.93304000 | 0.63776100 |
| H | -2.68181200 | 1.31546600 | 2.40200600 |
| H | -2.22105300 | -0.36842200 | 2.35184800 |
| H | -1.06975400 | 1.72754800 | 0.94159100 |
| H | 0.96110100 | 1.33614800 | 0.75144800 |
| H | 0.71107900 | -2.51969800 | -0.39975400 |
| H | 5.94331800 | -1.02461500 | 1.07212700 |
| H | 4.34787500 | -1.76878200 | 0.90604400 |
| H | 4.62911300 | -0.51297500 | 2.13443200 |
| H | 6.31560300 | 1.41033700 | 0.32781000 |
| H | 4.93895600 | 2.34701200 | -0.27298400 |
| H | 5.02493800 | 1.88875700 | 1.44026700 |
| H | 4.41313300 | 0.60353400 | -2.02980200 |
| H | 5.81061300 | -0.33482600 | -1.49665600 |
| H | 4.22134100 | -1.10528100 | -1.57139500 |
| H | -2.79926100 | 4.17761600 | -1.07567600 |
| H | -2.70910200 | 3.43742500 | 0.54670100 |
| H | -1.22335700 | 3.59980600 | -0.46858200 |

**17**

E= -900.1744174 a.u., number of negative frequencies = 0

1 1

| | | | |
|---|---|---|---|
| C | 0.02826300 | -0.28143600 | -0.58981900 |
| C | 0.25107100 | -0.76443000 | 0.82505900 |
| C | 1.38909700 | -1.51571500 | 1.38630000 |
| C | 2.64129100 | -2.01135500 | 0.72419000 |
| C | 2.74377100 | -1.93638700 | -0.80559700 |
| C | 3.09917900 | -0.54283800 | -1.37301700 |
| C | 2.44676800 | 0.69140700 | -0.72667200 |
| C | 0.93795400 | 0.91515000 | -0.97320400 |
| N | -1.37244600 | 0.11017900 | -0.75446400 |
| O | 0.10883300 | -2.28426500 | 0.93775900 |
| C | -2.44114700 | -0.59657300 | -0.25851600 |
| O | -3.57402300 | -0.17614500 | -0.81718900 |
| O | -2.36596900 | -1.46309300 | 0.60151100 |
| C | -4.80357200 | -0.76964600 | -0.32965100 |
| H | -0.60029800 | -2.48281900 | 1.58237200 |
| C | 0.48609600 | 2.20509000 | -0.28655700 |
| O | 0.15341600 | 3.20177400 | -0.86844100 |
| O | 0.53123000 | 2.08741600 | 1.05835500 |

| | | | |
|---|---|---|---|
| C | 0.16085000 | 3.27348400 | 1.81319800 |
| H | 0.26842100 | -1.10687400 | -1.26373800 |
| H | -0.36839000 | -0.27464000 | 1.56426500 |
| H | 1.43205800 | -1.49387600 | 2.47184900 |
| H | 2.77888500 | -3.04191400 | 1.06995900 |
| H | 3.45692000 | -1.44660600 | 1.19489100 |
| H | 3.53308700 | -2.62807800 | -1.10961300 |
| H | 1.83383900 | -2.34191100 | -1.25413700 |
| H | 4.17992100 | -0.40698100 | -1.26623100 |
| H | 2.90897800 | -0.54330500 | -2.45102300 |
| H | 2.95689200 | 1.57514400 | -1.11998800 |
| H | 2.64352700 | 0.70645000 | 0.34960100 |
| H | 0.78256400 | 1.08769000 | -2.04156300 |
| H | -1.58445500 | 0.75393800 | -1.50548100 |
| H | 0.26688100 | 2.99690100 | 2.85898500 |
| H | 0.82897300 | 4.09593700 | 1.56117200 |
| H | -0.86713300 | 3.55363300 | 1.58586000 |
| H | -4.92573900 | -0.55910800 | 0.73233500 |
| H | -5.59062000 | -0.29540200 | -0.90900600 |
| H | -4.79281700 | -1.84544000 | -0.49950700 |

**18**

E= -900.2096323 a.u., number of negative frequencies = 0

1 1

| | | | |
|---|---|---|---|
| C | 0.44166100 | -0.48617000 | -0.03527400 |
| C | -0.62351800 | -0.60979200 | -1.13470600 |
| C | -1.76606200 | -1.63568500 | -0.96542800 |
| C | -2.06404200 | -2.15106600 | 0.45141800 |
| C | -2.89342400 | -1.27864300 | 1.40895600 |
| C | -2.47082400 | 0.17586400 | 1.68747900 |
| C | -0.98957800 | 0.47226400 | 2.00426900 |
| C | -0.03404600 | 0.73765800 | 0.79412200 |
| N | 1.73855000 | -0.21250500 | -0.64574800 |
| O | -1.28834400 | -2.69896500 | -1.79087300 |
| C | 2.85200300 | -0.15894700 | 0.17963400 |
| O | 2.78381900 | -0.04172200 | 1.38447300 |
| C | -0.76858500 | 1.49790800 | -0.25110900 |
| O | -1.16943800 | 0.79261500 | -1.24583700 |
| O | 3.97151900 | -0.22780600 | -0.54424800 |
| C | 5.21637000 | -0.14046200 | 0.19292500 |
| O | -1.03596600 | 2.73839400 | -0.17338400 |
| C | -1.80537500 | 3.40953000 | -1.23989900 |
| H | 0.48380900 | -1.37948400 | 0.58737500 |
| H | -0.20001400 | -0.78544300 | -2.12113800 |
| H | -2.67085000 | -1.19123200 | -1.40325600 |

| | | | |
|---|---|---|---|
| H | -2.63306300 | -3.07794900 | 0.31793900 |
| H | -1.12914800 | -2.46474000 | 0.92289000 |
| H | -3.92969800 | -1.25246800 | 1.05637900 |
| H | -2.92234800 | -1.81480200 | 2.36253800 |
| H | -3.05492900 | 0.50827100 | 2.54954300 |
| H | -2.81617900 | 0.81936500 | 0.86838100 |
| H | -0.94552900 | 1.37259600 | 2.62028900 |
| H | -0.53638100 | -0.32548600 | 2.59861800 |
| H | 0.82525500 | 1.29544500 | 1.16144100 |
| H | 1.91243600 | -0.58599300 | -1.56951900 |
| H | -1.85468100 | -3.47239600 | -1.69277600 |
| H | 5.99529700 | -0.22576800 | -0.55961800 |
| H | 5.27979700 | 0.81747600 | 0.70822800 |
| H | 5.28295000 | -0.95469900 | 0.91356200 |
| H | -1.83026700 | 4.45006800 | -0.93476500 |
| H | -1.28233000 | 3.28053200 | -2.18508500 |
| H | -2.80416500 | 2.97936300 | -1.27616700 |

**19**

E= -900.1980382 a.u., number of negative frequencies = 0

1 1

| | | | |
|---|---|---|---|
| C | 0.28182700 | -1.02817400 | 0.50897500 |
| C | -0.74373100 | -1.89168700 | -0.28322200 |
| C | -1.71930000 | -1.03538500 | -1.15673000 |
| C | -3.14517800 | -0.73047200 | -0.61636900 |
| C | -3.41909700 | 0.70250800 | -0.12112200 |
| C | -2.80371200 | 1.11446200 | 1.24023600 |
| C | -1.54539200 | 0.35417200 | 1.67757100 |
| C | -0.28164400 | 0.40015100 | 0.74193100 |
| N | 1.54211200 | -0.99696000 | -0.23715700 |
| C | 2.66341000 | -0.32565100 | 0.21199800 |
| O | 2.64808000 | 0.58704000 | 1.01584000 |
| C | -0.50740100 | 0.94952400 | -0.62514600 |
| O | -1.04743700 | 0.23285000 | -1.54191300 |
| O | -1.53956300 | -2.69677900 | 0.56502700 |
| O | 3.75369800 | -0.80090900 | -0.39660400 |
| C | 5.00842200 | -0.14824400 | -0.08017400 |
| O | -0.13202400 | 2.09944300 | -1.04315800 |
| C | 0.56490700 | 3.06972300 | -0.18396200 |
| H | 0.41637700 | -1.48298800 | 1.49595400 |
| H | -0.18306400 | -2.53020300 | -0.97360300 |
| H | -1.81211800 | -1.52012700 | -2.12415900 |
| H | -3.83368000 | -0.92346600 | -1.44092000 |
| H | -3.37657300 | -1.46660200 | 0.15369400 |
| H | -4.50410800 | 0.80191400 | -0.04327300 |

| | | | |
|---|---|---|---|
| H | -3.13603500 | 1.41257900 | -0.90251800 |
| H | -3.54100500 | 0.94827200 | 2.03001100 |
| H | -2.62089300 | 2.19450800 | 1.23907400 |
| H | -1.22288000 | 0.73451200 | 2.64852500 |
| H | -1.80612700 | -0.69273300 | 1.83407600 |
| H | 0.49466200 | 0.99319000 | 1.21529700 |
| H | 1.75546600 | -1.80647200 | -0.80438900 |
| H | -1.10067300 | -3.53682700 | 0.73734000 |
| H | 5.75963700 | -0.69803400 | -0.64020500 |
| H | 4.97949700 | 0.89407600 | -0.39694600 |
| H | 5.20200800 | -0.20701000 | 0.98998300 |
| H | 0.76083300 | 3.90648400 | -0.84674200 |
| H | -0.09652700 | 3.36931700 | 0.62676700 |
| H | 1.48972400 | 2.62959100 | 0.18469400 |

**20**

E= -860.5560991 a.u., number of negative frequencies = 0

0 1

| | | | |
|---|---|---|---|
| C | 0.29221700 | 0.12578400 | 0.30011900 |
| C | -0.03003800 | -0.93354800 | -0.78342700 |
| C | -1.51370000 | -1.00137600 | -1.17913800 |
| C | -2.51513300 | -1.65439400 | -0.20916100 |
| C | -3.19903000 | -0.79088600 | 0.86367500 |
| C | -2.29807000 | -0.21520500 | 1.96703700 |
| C | -1.64879700 | 1.14060400 | 1.65741500 |
| C | -0.77783200 | 1.24111300 | 0.37607700 |
| N | 1.60989600 | 0.73840500 | 0.08072000 |
| C | 2.76714600 | 0.03801900 | 0.15685100 |
| O | 2.84891500 | -1.17308300 | 0.30350000 |
| C | -1.65433200 | 1.40088200 | -0.87123700 |
| O | -2.08975700 | 2.47601200 | -1.19679500 |
| O | -1.95031900 | 0.31170600 | -1.61065600 |
| O | 0.35686900 | -2.23847300 | -0.40425200 |
| O | 3.83716700 | 0.85399900 | 0.04623400 |
| C | 5.12335900 | 0.20739800 | 0.06446100 |
| H | 0.32856000 | -0.39955300 | 1.25819300 |
| H | 0.50149100 | -0.62092500 | -1.69378500 |
| H | -1.53793700 | -1.58940800 | -2.09700200 |
| H | -1.99797800 | -2.49815600 | 0.25756800 |
| H | -3.31024100 | -2.08369100 | -0.82620800 |
| H | -3.94696800 | -1.43571400 | 1.33565000 |
| H | -3.76823700 | 0.01522000 | 0.38709900 |
| H | -1.53444200 | -0.95488200 | 2.23422400 |
| H | -2.89545600 | -0.07385400 | 2.87362100 |
| H | -1.00827500 | 1.41994000 | 2.50109300 |

| | | | |
|---|---|---|---|
| H | -2.42354600 | 1.91042900 | 1.58874900 |
| H | -0.28016400 | 2.21103000 | 0.44429200 |
| H | 1.68315200 | 1.73638200 | -0.04503500 |
| H | 1.29321400 | -2.19180200 | -0.14473600 |
| H | 5.84760000 | 1.01185100 | -0.04168500 |
| H | 5.27273100 | -0.32070200 | 1.00658500 |
| H | 5.20949500 | -0.49662400 | -0.76394400 |

**TS14-15**

E= -1018.154972 a.u., im. freq.= 187.48*i*

1 1

| | | | |
|---|---|---|---|
| C | 0.26432900 | 0.06226600 | -0.33147400 |
| C | 1.09672400 | 0.77394100 | -1.35606100 |
| C | 1.61386900 | 2.14483800 | -1.19706100 |
| C | 1.60393800 | 2.87549100 | 0.12293600 |
| C | 2.81069100 | 2.58772200 | 1.02925900 |
| C | 3.08607300 | 1.10808900 | 1.34992400 |
| C | 1.87365100 | 0.25932300 | 1.80273000 |
| C | 1.17382900 | -0.63746700 | 0.73788800 |
| C | 2.21922400 | -1.43627500 | -0.01306300 |
| O | 2.63503300 | -1.05585800 | -1.10033900 |
| N | -0.63657100 | -0.85632000 | -0.99365400 |
| C | -1.99376000 | -1.00527100 | -0.71072700 |
| O | -2.64421200 | -1.89336400 | -1.20457100 |
| O | -2.41202300 | -0.05042200 | 0.13002100 |
| C | -3.85288700 | 0.04417400 | 0.55086500 |
| H | -0.35666000 | 0.78894200 | 0.19164300 |
| H | 1.25488500 | 0.30581600 | -2.31615700 |
| H | 2.43142400 | 2.41327300 | -1.86340000 |
| H | 1.57053300 | 3.94434200 | -0.10705200 |
| H | 0.67086300 | 2.64927400 | 0.64352300 |
| H | 3.71364700 | 3.02336900 | 0.59018600 |
| H | 2.63645000 | 3.13521500 | 1.95987300 |
| H | 3.82942700 | 1.09029500 | 2.15034700 |
| H | 3.57975400 | 0.63564700 | 0.49533000 |
| H | 2.20750800 | -0.43075600 | 2.58073000 |
| H | 1.11006800 | 0.88509500 | 2.27558300 |
| H | 0.51120500 | -1.31629600 | 1.27851300 |
| H | -0.25732300 | -1.63283500 | -1.51807800 |
| C | -3.84040100 | 1.26210700 | 1.47079200 |
| H | -4.84719400 | 1.44413600 | 1.85213900 |
| H | -3.17839900 | 1.10044200 | 2.32477200 |
| H | -3.51411300 | 2.15518600 | 0.93273100 |
| C | -4.24277800 | -1.22107700 | 1.31249000 |
| H | -5.24979900 | -1.09550500 | 1.71781000 |

| | | | |
|---|---|---|---|
| H | -4.23931900 | -2.09648600 | 0.66519500 |
| H | -3.56392600 | -1.39180400 | 2.15201700 |
| C | -4.72469500 | 0.28836600 | -0.67923100 |
| H | -4.72792300 | -0.57203500 | -1.34651800 |
| H | -5.75119000 | 0.47697400 | -0.35545600 |
| H | -4.38027300 | 1.16847000 | -1.22834900 |
| O | 2.64805700 | -2.50526300 | 0.62036100 |
| C | 3.69866900 | -3.29314600 | -0.01284500 |
| H | 3.88586600 | -4.11237200 | 0.67460800 |
| H | 3.34993500 | -3.66306200 | -0.97588600 |
| H | 4.58939400 | -2.68056100 | -0.14479700 |
| O | 0.28379500 | 2.31559300 | -1.85925600 |
| H | 0.37710500 | 2.40551100 | -2.82197600 |

**TS14-16**

E= -1018.1380698 a.u., im. freq.= 245.47*i*

1 1

| | | | |
|---|---|---|---|
| C | -0.51294200 | -0.22557100 | 0.62493800 |
| C | -0.70728200 | -1.22665100 | -0.50492800 |
| C | -1.96321800 | -1.91583200 | -0.77002700 |
| C | -2.99950400 | -2.27671600 | 0.21254100 |
| C | -4.23465000 | -1.32815200 | 0.06599900 |
| C | -4.17831300 | 0.02338700 | 0.83040400 |
| C | -2.88299900 | 0.48137700 | 1.53031600 |
| C | -1.62079200 | 0.87204900 | 0.68332400 |
| N | 0.78755900 | 0.43053600 | 0.46430000 |
| C | 1.95694100 | -0.23164600 | 0.11495200 |
| O | 2.99872200 | 0.52871500 | 0.42416200 |
| O | 1.98282000 | -1.32760300 | -0.42388600 |
| C | -2.05381700 | 1.31688100 | -0.71589900 |
| O | -2.35620700 | 0.49227200 | -1.55830600 |
| O | -0.35180500 | -2.60940000 | -0.15774500 |
| C | 4.41483100 | 0.15524800 | 0.07133100 |
| C | 4.79416500 | -1.12862100 | 0.80567300 |
| C | 5.19878400 | 1.35151600 | 0.60278900 |
| C | 4.54240800 | 0.03653100 | -1.44559000 |
| O | -2.17615800 | 2.60444800 | -1.01896500 |
| C | -1.82821200 | 3.67589800 | -0.11414500 |
| H | -0.55024500 | -0.77898900 | 1.57135100 |
| H | -0.20444600 | -0.91463800 | -1.41203600 |
| H | -2.10764100 | -2.22766600 | -1.80012800 |
| H | -3.33163500 | -3.29353400 | -0.01058400 |
| H | -2.61049600 | -2.25951000 | 1.23004500 |
| H | -5.08624200 | -1.90488200 | 0.42996900 |
| H | -4.42136900 | -1.14822400 | -0.99362200 |

| | | | |
|---|---|---|---|
| H | -4.92936600 | -0.01548700 | 1.62394800 |
| H | -4.51540600 | 0.81244100 | 0.15361500 |
| H | -3.15995800 | 1.36977400 | 2.10382400 |
| H | -2.57079100 | -0.25042000 | 2.28135400 |
| H | -1.15783200 | 1.70423600 | 1.21368600 |
| H | 0.94747600 | 1.24272600 | 1.04405500 |
| H | 0.41387000 | -2.85824900 | -0.70929900 |
| H | 5.85783600 | -1.32472900 | 0.65039200 |
| H | 4.23074100 | -1.98516500 | 0.43849000 |
| H | 4.62741700 | -1.02211100 | 1.88024400 |
| H | 6.26383100 | 1.20464000 | 0.41179000 |
| H | 4.88564600 | 2.27366100 | 0.10904600 |
| H | 5.05599900 | 1.46373800 | 1.67945500 |
| H | 4.19746300 | 0.95051700 | -1.93510400 |
| H | 5.59520500 | -0.10288000 | -1.70251700 |
| H | 3.98154300 | -0.81279300 | -1.83336400 |
| H | -2.09442400 | 4.58545700 | -0.64632300 |
| H | -2.40502800 | 3.61590800 | 0.81002200 |
| H | -0.75642400 | 3.67677800 | 0.08945100 |

**TS17-18**

E= -900.1711102 a.u., im. freq.= 192.95*i*

1 1

| | | | |
|---|---|---|---|
| C | 0.50116000 | -0.11064500 | -0.11789000 |
| C | -0.19909300 | -0.79946400 | -1.25228900 |
| C | -0.80136300 | -2.14147200 | -1.15749900 |
| C | -1.03138500 | -2.84560900 | 0.15708800 |
| C | -2.34562100 | -2.48320400 | 0.86538800 |
| C | -2.59126900 | -0.98700800 | 1.12883500 |
| C | -1.41942400 | -0.18858800 | 1.74878800 |
| C | -0.52364500 | 0.65295600 | 0.79034200 |
| C | -1.40466000 | 1.47951200 | -0.12375600 |
| O | -1.66168100 | 1.09379100 | -1.25775200 |
| N | 1.53871300 | 0.75416200 | -0.64231800 |
| C | 2.84154200 | 0.82441800 | -0.17273800 |
| O | 3.63165300 | 1.65010100 | -0.55165600 |
| O | 3.08867300 | -0.14528400 | 0.72980700 |
| C | 4.44177700 | -0.17737300 | 1.25175200 |
| H | 0.99514400 | -0.85847600 | 0.50130100 |
| H | -0.18064500 | -0.34496600 | -2.23145700 |
| H | -1.51839300 | -2.38436200 | -1.93933500 |
| H | -1.01799900 | -3.91892000 | -0.05330200 |
| H | -0.17802400 | -2.65664400 | 0.81215500 |
| H | -3.19239400 | -2.88070200 | 0.29725000 |
| H | -2.34320200 | -3.02391400 | 1.81601200 |

| | | | |
|---|---|---|---|
| H | -3.44236900 | -0.92128300 | 1.81061200 |
| H | -2.93053000 | -0.50538000 | 0.20678100 |
| H | -1.83128000 | 0.53281900 | 2.45784100 |
| H | -0.76805500 | -0.83977300 | 2.34030000 |
| H | 0.08004500 | 1.31317900 | 1.41634600 |
| H | 1.28526000 | 1.53614300 | -1.23042100 |
| O | -1.88111000 | 2.57569700 | 0.42089400 |
| C | -2.78878100 | 3.39237200 | -0.37684800 |
| H | -3.04430900 | 4.23123300 | 0.26321600 |
| H | -2.28129700 | 3.72945500 | -1.27941600 |
| H | -3.67345900 | 2.81247100 | -0.63575100 |
| O | 0.60496100 | -2.38811100 | -1.60219100 |
| H | 0.65779800 | -2.49632200 | -2.56620300 |
| H | 4.45552400 | -1.00537000 | 1.95567100 |
| H | 5.15054100 | -0.34530800 | 0.44188400 |
| H | 4.67126200 | 0.76205200 | 1.75287600 |

**TS17-19**

E= -900.1540246 a.u., im. freq.= 242.61*i*

1 1

| | | | |
|---|---|---|---|
| C | 0.34242500 | -0.18230600 | 0.63365600 |
| C | 0.21881200 | -1.19340800 | -0.49783000 |
| C | -0.99470300 | -1.95240600 | -0.76943100 |
| C | -2.01706900 | -2.36557500 | 0.20675800 |
| C | -3.30326500 | -1.49015400 | 0.04412500 |
| C | -3.32997800 | -0.13077000 | 0.79632900 |
| C | -2.07117400 | 0.40262000 | 1.50923100 |
| C | -0.82185500 | 0.85624500 | 0.67421300 |
| N | 1.61109200 | 0.54003600 | 0.49582400 |
| C | 2.80655100 | -0.05214300 | 0.14217700 |
| O | 3.82129700 | 0.76042800 | 0.44291000 |
| O | 2.92023800 | -1.13853800 | -0.39812100 |
| C | -1.26400000 | 1.27127200 | -0.73166200 |
| O | -1.50704200 | 0.42702100 | -1.57391700 |
| O | 0.64886200 | -2.55304900 | -0.14292700 |
| C | 5.14518600 | 0.31643900 | 0.05169100 |
| O | -1.45947200 | 2.54781700 | -1.03979200 |
| C | -1.18702200 | 3.64149100 | -0.13501100 |
| H | 0.32152300 | -0.73554800 | 1.58023500 |
| H | 0.71045900 | -0.85503000 | -1.40169700 |
| H | -1.11289300 | -2.27624600 | -1.79921400 |
| H | -2.28904800 | -3.40083100 | -0.01308300 |
| H | -1.63892500 | -2.32094000 | 1.22751700 |
| H | -4.12234900 | -2.11203800 | 0.40825400 |
| H | -3.49343300 | -1.33080300 | -1.01821600 |

| | | | |
|---|---|---|---|
| H | -4.08878900 | -0.20319800 | 1.58001000 |
| H | -3.69971000 | 0.63317800 | 0.10794400 |
| H | -2.40355300 | 1.27883700 | 2.07183200 |
| H | -1.72867300 | -0.30499700 | 2.27014000 |
| H | -0.40984700 | 1.71426600 | 1.20563300 |
| H | 1.72075800 | 1.36345300 | 1.07175400 |
| H | 1.42001400 | -2.77354300 | -0.69835500 |
| H | -1.50129700 | 4.53149600 | -0.67389400 |
| H | -1.76996800 | 3.54967300 | 0.78262800 |
| H | -0.11958600 | 3.70773500 | 0.08034400 |
| H | 5.19690000 | 0.20373400 | -1.03068900 |
| H | 5.81474400 | 1.10239000 | 0.38950300 |
| H | 5.38152900 | -0.62909500 | 0.53789400 |

## References

1. Kardos, M.; Kiss L.; Fülöp, F. *Asian J. Org. Chem*. **2015**, 4, 1155-1159.

2. Karavaizoglu, U. N.; Salamci, E. *New J. Chem*. **2020**, *44*, 17976-17983.

3. Rigaku/MSC, Inc., 9009 new Trails Drive, The Woodlands, TX 77381-5209, USA, 2005.

4. G.M. Sheldrick, SHELXS-97, SHELXL-97 Program for Crystal Structure Solution and refinement, University of Gottingen, Göttingen, Germany, 1997.
